# Revisiting the therapeutic potential of tocotrienol

**DOI:** 10.1002/biof.1873

**Published:** 2022-06-20

**Authors:** Ranmali Ranasinghe, Michael Mathai, Anthony Zulli

**Affiliations:** ^1^ Institute of Health and Sport, College of Health and Medicine Victoria University Melbourne Victoria Australia

**Keywords:** antioxidant behavior, cancer studies, chemotherapy, disease pathology, tocotrienol in cancer immunology, tocotrienols, vitamin E

## Abstract

The therapeutic potential of the tocotrienol group stems from its nutraceutical properties as a dietary supplement. It is largely considered to be safe when consumed at low doses for attenuating pathophysiology as shown by animal models, in vitro assays, and ongoing human trials. Medical researchers and the allied sciences have experimented with tocotrienols for many decades, but its therapeutic potential was limited to adjuvant or concurrent treatment regimens. Recent studies have focused on targeted drug delivery by enhancing the bioavailability through carriers, self‐sustained emulsions, nanoparticles, and ethosomes. Epigenetic modulation and computer remodeling are other means that will help increase chemosensitivity. This review will focus on the systemic intracellular anti‐cancer, antioxidant, and anti‐inflammatory mechanisms that are stimulated and/or regulated by tocotrienols while highlighting its potent therapeutic properties in a diverse group of clinical diseases.

**Abbreviations**
5‐FU: 5‐fluorouracil
ABC transporters: ATP‐cassette binding protein family
*ADCY1*
: adenylyl cyclase type 1.
AKI: acute kidney injury
Akt: protein kinase‐B
alpha TTP: alpha‐tocopherol transfer protein
AMPK: adenosine mono phosphate kinase
Ang‐1: angiopoietin‐1
ATF3: activating transcription factor‐3
Bcl‐2: B‐cell lymphoma 2
Bcl‐xL: B‐cell lymphoma‐extra‐large
BMDM: bone marrow‐derived macrophages
*BSG*
: basigin‐encoding plasma membrane protein in spermatogenesis
CAC: colitis‐associated colorectal cancer
*CD3‐epsilon*
: cluster of differentiation 3‐epsilon
CDK 1, CDK2, CDK4, CDK6: cyclin‐dependent kinase 1, 2, 4, and 6
cDNA: complimentary deoxyribonucleic acid
*CFLAR*
: apoptosis regulator
CNS: central nervous system
COPD: chronic obstructive pulmonary disorder
COX‐2: cyclooxygenase‐2
CSC: cancer stem cells
CVD: cardiovascular disease
DAMP: damage‐associated molecular patterns
DCs: dendritic cells
DHA: docosahexaenoic acid
DKD: diabetic kidney disease
DR5: death receptor 5
DSS: dextran sulfate sodium
EGFR: epidermal growth factor receptor
EMT: epithelial‐to‐mesenchymal transition
ER: endoplasmic reticulum
ERK1 and ERK2: extracellular signal‐regulated protein kinase 1 and 2
FasL: Fas ligand
*FGF18*
: fibroblast growth factor 18
FLICE: Fas‐associated death domain‐like IL‐1‐converting enzyme
G6PD: glucose‐6‐phosphate dehydrogenase
G‐CSF: granulocyte colony‐stimulating factor
GnRH: gonadotropin‐releasing hormone
GSSG: glutathione disulfide
HCC: hepatocarcinoma cell line
HDL: high‐density lipoprotein
HIF‐1 α: hypoxia‐inducible 1‐alpha
HMG‐CoA: 3‐hydroxy 3‐methyl glutaryl coenzyme A
HPLC: high‐pressure liquid chromatography
IDL: intermediate‐low‐density lipoprotein
IL‐6: interleukin‐6
iNOS: inducible nitric oxide synthase
Insigs: insulin‐induced genes
Jak/stat‐3/6: Janus kinase/signal transducer and activator of transcription proteins‐induce cellular senescence
JNK: c‐Jun N‐terminal kinase
K562 cells: chronic myeloid leukemia
LDL: low‐density lipoprotein
LH: luteinising hormone
LPS: lipopolysaccharide
MAPK: mitogen‐activated protein kinase
MCF‐7: Michigan Cancer Foundation‐7
MDA: malondialdehyde
MDA‐MB‐231: Anderson‐metastatic breast 231
MDR‐1: multidrug‐resistant protein‐1
MDRSM: mixture design response surface methodology
miR: microribonucleic acid
MMP‐2 and MMP‐9: matrix metalloproteinase‐2 and ‐9
mTOR: mammalian target of rapamycin
NF‐kB/p65: nuclear factor kappa beta/protein 65
*NKX3‐1*
: androgen‐regulated homeobox gene‐1
NLRP3: nod‐like receptor protein‐3
Nox‐2: NADPH oxidase
Nrf2: nuclear factor‐erythroid factor 2‐related factor 2
NSCLC: nonsmall cell lung cancer
OPG: osteoprotegerin
p‐eIf2α: translation initiation factor 2
p16: protein 16
p21: protein 21
p27: protein 27
p38: protein 38
p53: protein 53
PARP: poly‐ADP‐ribose polymerase
PCa: prostate cancer
PD: Parkinsonian disease
PERK/eIF2alpha/ATF‐4: activation‐mediated protein kinase‐like ER kinase/eukaryotic translational initiation factor/activating transcription factor 4
P‐gp: P‐glycoprotein
PI3K/Akt: phosphoinositide‐3‐kinase/protein kinase B
p‐JNK: jun N‐terminal kinase
PSP: polysaccharopeptide
PUFA: poly unsaturated fatty acids
RA: rheumatoid arthritis
Raf–ERK: rapidly accelerated fibrosarcoma–extracellular signal‐regulated kinases
RANKL: receptor activator of nuclear factor‐kappa‐Β ligand
Ras: rat sarcoma‐derived guanine nucleotide‐binding protein
RIPI: receptor‐interacting protein serine/threonine kinase
ROS: reactive oxygen species
RSK: ribosomal protein S6 kinase
SCC: squamous cell carcinoma
SHP1 and SHP2: Src homology 2 domain‐containing protein tyrosine phosphatase 1 and 2
siRNA: small interfering ribonucleic acid
SIRT‐1: sirtuin 1
*SNX9*
: sorting nexin 9
SOD: superoxide dismutase
STAT3: signal transducer and activator of transcription 3
TAK1: activated kinase 1
TGF‐β: transforming growth factor‐β
TGF‐β: transforming growth factor‐beta
TMP1 and TMP2: tissue metalloproteinase 1 and 2
TNM: tumor, nodes, and metastases
*TP53*
: tumor protein 53
TRAIL: TNF‐alpha‐related apoptosis inducing‐ligand
TRF: tocotrienol rich fraction
UACR: urine albumin to creatinine ratio
uPA: urokinase‐type plasminogen activator
VEGF: vascular endothelial growth factor
VLDL: very‐low‐density lipoproteins
γ‐CEHC: gamma‐carboxyethyl hydroxychromanol

## 1 INTRODUCTION

### 1.1 What are tocotrienols?

**FIGURE 1:**
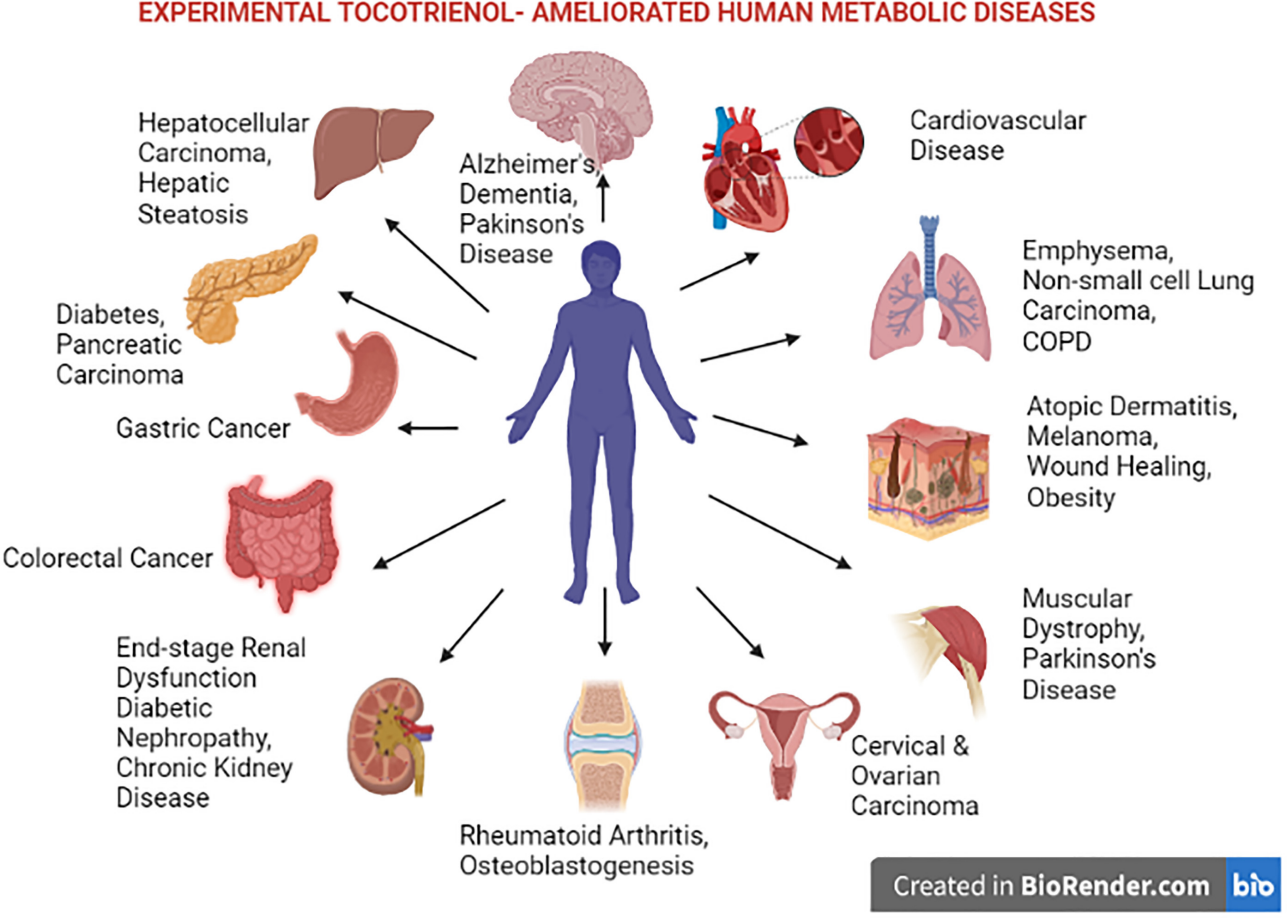
Human diseases attenuated by experimental tocotrienol treatment

Tocotrienols and tocopherols are two groups of vitamin E^1^ which possess considerable therapeutic potential owing mostly to their anticancer,^2^ antioxidant,^3^ anti‐diabetic,^4^ anti‐inflammatory,^5^ nephroprotective,^6^ neuroprotective,^7^ hepatoprotectives,^8^ and cardiovascular‐protective properties.^9^ These different forms of vitamin E can only be obtained from plants^10^ and are known to be the principal fat‐soluble, antioxidants present in the human blood plasma.^11^ Thus, they show promise in attenuating a spectrum of human diseases (Figure 1).^12^ These are dietary components which can be transported due to lipophilic nature^13^ and their nutraceutical value has bestowed a beneficial paradigm of clinical attributes in both animal models and human studies.^14^ The use of vitamin E compounds dates to the late 19th century when the tocopherols were first identified as having medicinal effects.^15^ Early studies were focused more on tocopherol benefits but the current trend is to evaluate the therapeutic potential of tocotrienols in clinical research,^16^ with emphasis on biological functions performed by this group including cellular proliferation, cell differentiation, cellular regeneration, repair mechanisms, apoptosis, and autophagy.^17^ Tocotrienols have gained increasing attention with the discovery of being a novel candidate for noncommunicable disease therapy and are evaluated in clinical trials.^18^, ^19^

### 1.2 Chemical structure and sources

**FIGURE 2:**
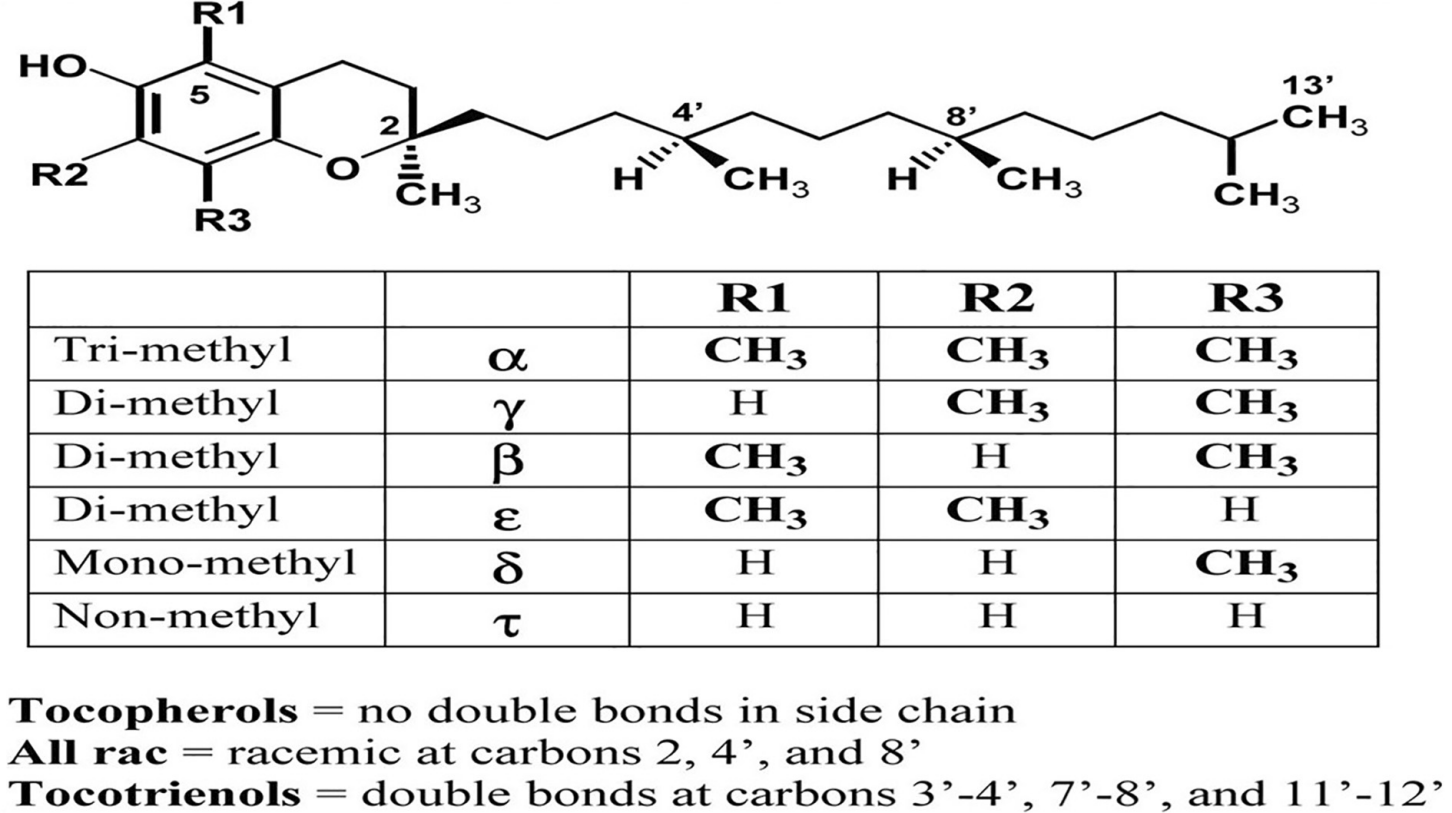
Stereochemical structure of tocotrienols and tocopherols^314^

The chemical structure of vitamin E was elucidated in 1938 by Fenholz.^20^ The two main forms of vitamin E, the tocopherols and tocotrienols, comprise four sub‐forms each (named alpha, beta, gamma, and delta) and share structural similarity^21^ except for a few differences that account for functional modifications. The tocotrienol sub‐forms offer superior therapeutic potential compared to the tocopherol counterparts.^22^, ^23^ In addition to the eight different forms of vitamin E that are established, two more variants named des‐methyl and di‐des‐methyl tocotrienols have been added to this vitamer profile.^24^ This eight‐member vitamin E family shares a chromanol head having a hydrocarbon tail attached at the 2‐position of the ring. The tocopherol group consists of a saturated, phytyl side‐chain whereas tocotrienol has an unsaturated isoprenoid side chain comprising of three *trans*‐carbon–carbon double bonds (Figure 2).^25^ The four different forms of each differ in their R1 and R2 groups on the chromanol ring substituted with either a methyl group or hydrogen atoms.^26^ There is evidence that the tocotrienols have a better ability to diffuse through the phospholipid bilayer of the cell membrane because of their unsaturated isoprenoid tail compared to the phytyl tail of tocopherol.^27^ Their lipid‐solubility and hydrophobicity have required membrane contact or specific protein carriers for its cellular transportation which occurs in plasma in association with plasma phospholipid transfer proteins.^28^ Out of the eight different forms of vitamin E, alpha‐tocopherol was identified before tocotrienols^29^ but with the progress of research, tocotrienols became recognized as superior to RRR‐alpha‐tocopherol in their ability to penetrate the blood–brain barrier and the liver because of their small size and lipophilic nature.^30^ The tocotrienol therapeutic efficacy was overshadowed by the tocopherols until recent years during which the tocotrienols were recognized as being more potent in treating a wide range of diseases thus playing a prominent role in health and disease. As such, more preclinical and clinical studies are needed to improve its medicinal properties which are confirmed as being more curative than the tocopherol forms which were thought to be more therapy‐wise superior to the tocotrienols. An array of other different synthetic vitamin E forms exist, such as the tocopherol‐member family that includes the acetate and succinate forms of tocopherols, and the phosphorylated tocopherols in addition to the four principal forms of tocopherols—alpha, beta, gamma, and delta. Early research showed that treatment with alpha‐tocopherol and alpha‐tocopheryl acetate was suppressive on mitomycin‐stimulated—human Tenon's fibroblast proliferation.^31^

The succinate form of alpha‐tocotrienol was more effective than the aforementioned two vitamin‐E forms in suppressing restimulated fibroblasts via a decrease in glucose‐6‐phosphate dehydrogenase (G6PD) activity and upregulated necrosis.^31^ Another vitamin E‐form is tocopheryl phosphate which is involved in gene regulation, inhibition of cell proliferation, and limiting lipid accumulation in cells.^32^ An antagonistic effect between alpha‐tocopherol and alpha‐tocopheryl phosphate on the phosphoinositide‐3‐kinase/protein kinase B (PI3K/Akt) pathway was observed and the stimulation of the PI3Kγ/Akt pathway by alpha‐tocopheryl phosphate leads to the induction of vascular endothelial growth factor (VEGF) expression.^32^ Additionally, the redox‐inactive tocotrienol (6‐*O*‐carboxypropyl‐α‐tocotrienol) was tested against malignant mesothelioma, which is an aggressive form of cancer that has poor prognosis, that produced enhanced anti‐cancer effects than with tocotrienol given alone.^33^ Further, its oral administration in a xenografted mouse model augmented anti‐tumor effects without the loss of body weight, than the normal tocotrienols administered through the same route.^33^ Vitamin E was first identified in 1922 from a green leafy vegetable extract^34^ where the ill effects of ingesting rancid fat were thus overcome by consuming it and research which had ensued thereafter had designated it an essential vitamin.^35^ Vitamin E is synthesized exclusively by all photosynthetic organisms. The biosynthesis of tocotrienols occurs in the seed endosperm of most monocotyledons, for example, cereal crops such as wheat, barley, and rice, and in some dicotyledons (families Apiaceae and Solanaceae, which includes Tobacco).^36^, ^37^ Crude palm oil extracts are known to contain up to 800 mg/kg of tocotrienol, obtained particularly from the fruits of *Elaeis guineensis*, commonly known as the African oil palm or Macaw‐fat, which is the principal source of palm oil.^38^ Palm oil is a major source of tocotrienol which is extracted from the palm fruit mesocarp, the endosperm, and kernel oil, and contains up to 30% of tocopherol and 70% of tocotrienol comprising alpha, gamma, and delta forms as the principal constituents).^39^ It also consists of 50% saturated fatty acids, 40% of unsaturated fatty acids and 10% of polyunsaturated fatty acids unlike any other edible plant or animal oil^40^ and is devoid of trans fatty acids.^41^ Gamma‐tocotrienol is the abundant and active natural form of tocotrienol present in rice bran oil (*Oryza sativa*) which consists of antioxidant activity^42^ although in oats (*Avena sativa*) the active ingredient is alpha‐tocotrienol.^43^ Hulled and dehulled wheats (*Triticum spp*.,) contain beta‐tocotrienol as its natural source of tocotrienol,^44^ although it is doubted whether the consumption of these foods provides an adequate supply of tocotrienol that is required to maintain good health in the human populations. The biologically effective doses have been described to be between 100–200 g for palm oil or rice bran oil and 1.5–4 kg are needed from wheat‐germ, barley, or oats where notably a 1000 kg of crude palm oil is needed to derive 1 kg of a commercially produced tocotrienol and tocopherol supplementation, named Tocomin (containing 50% of tocopherols and tocotrienols) which points out that the consumption of commercial preparations are more beneficial than consuming natural sources of tocotrienols.^15^ Tocotrienol is also synthesized by annatto (*Bixa orellana*) seeds^45^ and nonfood plants such as rubber (*Hevea brasiliensis*) and is contained in the rubber latex.^46^

### 1.3 Genetic basis of tocotrienol action

Genetic studies by a clinical trial using peripheral blood mononuclear cells have identified the putative genes that are associated with the tocotrienol therapeutic action. This gene profile consisted of the cluster of differentiation 3‐epsilon *(CD3‐epsilon)*, sorting nexin 9 (*SNX9*), tumor protein 53 *(TP53)*, androgen‐regulated homeobox gene‐1 (*NKX3‐1)*, apoptosis regulator (*CFLAR)*, basigin‐encoding plasma membrane protein in spermatogenesis (*BSG)*, fibroblast growth factor 18 (*FGF18)*, matrix metalloproteinase‐7 *(MMP7)*, mitochondrial creatine kinase‐2 (*CKMT2)*, and adenylyl cyclase type 1 (*ADCY1)*.^47^ When treatment was continued for 3 months with the tocotrienol rich fraction (TRF), it produced the upregulation of the gene expression of; cell division, transcription, G protein‐coupled receptor signaling, multicellular organismal growth, protein kinase activity, cell surface receptor signaling, and response to glucocorticoids with the downregulation of the aging‐related genes.^47^ Additionally, the modulation of biological functions through gene expressions has been reported. They included downregulating G‐protein coupled receptor signaling, phosphoinositide, and integrin‐mediated signaling, apoptotic processes, cell–cell signaling, extracellular signal‐regulated protein kinase 1 and 2 (ERK1 and ERK2) signaling cascade, cell surface receptor signaling, and stimulating multicellular organismal growth, when treated with TRF for 6 months.^47^

Gene expression analysis of MCF‐7 cells treated with gamma‐tocotrienol revealed alterations in the expression of multiple genes involved in cell growth and proliferation, cell death, cell cycle, cellular development, cellular movement, and gene expression. Further analysis of differentially modulated genes using Ingenuity Pathway Analysis software suggested modulation of canonical signal transduction or metabolic pathways such as nuclear factor‐erythroid factor 2‐related factor 2 (Nrf2)‐mediated oxidative stress response, transforming growth factor‐beta (TGF‐β) signaling and Endoplasmic Reticulum (ER) stress response by the different forms of tocotrienol.

### 1.4 Tocotrienol metabolism

The major drawback of tocotrienol therapy is that it is metabolized and excreted rapidly from the body.^48^ It is either removed after solubilization in bile acids and egested in feces or excreted in urine after shortening of the side chains to make it more water‐soluble.^49^ The bioavailability is of short duration and is not sustained in plasma for an adequate period of time, which is not more than 3.5–4 h at which the tocotrienol concentration in the blood reaches the peak, but it completely disappears from blood after 24 h.^50^ An investigation into quantifying the tocotrienol level in nonsupplemented adult plasma has revealed a very low concentration of the common metabolite, gamma‐carboxyethyl hydroxychromanol (γ‐CEHC) to be 307 nM/128 μg L^−1^ although it is more than what was previously reported.^51^ Tocotrienol is hydrophobic, so requires protein carriers to enter a cell and is absorbed into the enterocytes lining the upper part of the small intestine and similar to other dietary lipids, are incorporated into chylomicrons that enter the blood plasma from the lymphatics.^52^ Vitamin E metabolism is controlled by the liver where its uptake, secretion, and transport occur via the alpha‐tocopherol transfer protein (alpha TTP) for which tocotrienols have a greater affinity.^53^ Absorption of tocotrienols is enhanced when consumed together with fat, to increase the bile secretion and because it protects poly unsaturated fatty acids (PUFA) from lipid peroxidation, it is delivered more to the sites of PUFA that is present in the body. Tocotrienols are metabolized in the liver, which is the nexus of vitamin E metabolism, where it undergoes omega‐hydroxylation by the cytochrome P450 activity followed by beta‐oxidation, conjugation, and excretion.^53^ Research has shown the involvement of the cellular senescence regulator, Sirtuin 1 (SIRT‐1) which delays senescence in cells playing a role in the cellular uptake and metabolism of tocotrienols.^50^ Tocotrienols show differential bioavailability in various organs such as the skin, brain, adipose tissue, muscles, cardiac muscle fiber, and liver suggesting the existence of another intracellular transport mechanism, that is independent of the alpha TTP carrier.^54^ However, among the different forms of tocotrienol, delta‐tocotrienol uptake was the highest followed by gamma‐tocotrienol and then alpha‐tocotrienol, in a study that utilized human diploid fibroblasts.^53^ Recent research has revealed preferential uptake and metabolism of tocotrienol forms, raising the need to identify the mechanisms for its intracellular augmentation and delivery to sites that otherwise hinders the therapeutic benefit of tocotrienols.

## 2 ANTI‐CANCER ACTIVITY OF TOCOTRIENOL

The quest for discovering a potential treatment strategy for cancer is ongoing (Table 1) as existing therapies have limited benefits. The conventional anti‐cancer treatment including chemotherapy, radiotherapy, and surgery are presented with many side effects and hence, novel drugs are needed which could promote bioavailability (the half‐maximal inhibitory concentration or IC50) and systemic efficacy (reaching and alleviating the disease states in different organs, for an example metastasis), while minimizing toxicity in cancer patients.^55^ In recent years, gamma and delta forms of tocotrienol have shown promising therapeutic efficacy in targeting common metabolic pathways^56^ to inhibit tumor progression (invasion, proliferation, angiogenesis, and metastasis) and cell cycle activity (through DNA damage and arresting G1/S phase of the cell cycle).^57^ They also promote the mechanisms of autophagy and apoptosis (via the caspase‐dependent and caspase‐independent pathways).^58^ Tocotrienols are more potent than the tocopherols in their ability to precisely target the cancerous cells from the noncancerous cells by the combination of a hypomethylated chroman ring (such as the alpha form that is fully methylated and delta form being the least methylated) and the isoprenoid tail that could evoke a well‐defined response of bioavailability and transformation.^59^ Data using breast and prostate cancer cells treated with gamma‐tocotrienols have revealed the destruction of the cancerous cells leaving the epithelial noncancerous cells unaffected.^60^ It makes tocotrienols superior anti‐neoplastic compounds^61^ as the conventional therapies induce nonspecific cellular destruction, causing side effects.^62^ Furthermore, gamma and delta tocotrienols are considered to be more potent anti‐cancer therapeutics compared to the other familial forms, in mediating cell death in research studies conducted with in vitro cell lines sourced from cancerous cervical,^63^ lung,^64^ breast,^65^ colon,^66^ liver,^67^ skin,^68^ prostate,^56^ blood,^69^ pancreas,^70^ gastric,^71^ and brain tissue^72^ (Figure 4). The anticancer pharmacokinetic action of tocotrienol is thought to increase with neoadjuvant combined therapy^73^ and several clinical trials are continuing at present.^18^, ^74^ Gamma‐tocotrienol in combination with a single dose of chemotherapy each with docetaxel (at concentrations of 0.5, 1, and 2 nM with 50, 75, and 100 μM for 6 days in an in vitro assay),^75^ cyclophosphamide (dosage not found) with a T3 mixture (Malaysian Palm Oil Board, Malaysia) that was composed of 27.45% alpha‐tocotrienol, 57.99%, gamma‐tocotrienol, and 14.56% delta‐tocotrienol, diluted in corn oil to the desired concentration^76^ are expected to induce anti‐cancer effects in an ongoing clinical trial. Additionally, epirubicin [at 0.33 mg/g and tocotrienol at 750 mg/g:tocotrienol mixture containing total tocotrienols 75% (750 mg/g); delta‐tocotrienol 89%; gamma‐tocotrienol and other tocotrienols 11%; Tocopherols 1% maximum],^77^ erlotinib (gamma‐tocotrienol at 0–4 μM and erlotinib at 0–5 μM),^78^ or ertuzumab (with gamma‐tocotrienol, delta‐tocotrienol and the synthetic derivative alpha‐tocopheryl polyethylene glycol 1000 succinate^73^ are under investigation in breast cancer patients in Denmark. Another human phase II trial is being conducted to determine the tocotrienol effect on ameliorating the side effects of the cancer treatment, FOLFOXIRI (5‐fluorouracil, oxaliplatin, and irinotecan).^79^ The first anti‐cancer drug to have experimented with in tocotrienol adjuvant therapy, was the breast cancer drug, tamoxifen which was unsuccessful with the researchers concluding that estrogen receptor‐positive‐early stages of breast cancer [of tumor, nodes, and metastases (TNM) Stages I and II] remained unresponsive to the tocotrienol‐rich‐fraction (TRF) administered with tamoxifen.^80^ However, the delta form of tocotrienol displayed efficacy in attenuating pancreatic ductal carcinoma (reaching adequate bioavailability levels in blood with significant neoplastic cell apoptosis) in a cohort of 25 persons given a dose of 200–1600 mg daily for 2 weeks before performing surgery. This highlights the usefulness of tocotrienol in pancreatic neoplasia, warranting further investigations.^81^ The adequate bioavailability was displayed by the IC_50_ of tumor cell growth inhibition, which was 40, 45, and 60 μM for delta, gamma, and beta tocotrienol compounds, respectively. The IC_50_ of growth inhibition for gemcitabine was 20 μM. In contrast alpha‐tocotrienol, alpha‐tocopherol, and delta‐tocopherol had no growth inhibitory activity. Malignant transformation was inhibited by 70, 30, 19, and 6% with delta, gamma, beta, and alpha tocotrienol compounds, respectively in MiaPaca‐2 cells. Malignant transformation inhibition for gemcitabine was 89%.^81^ In this study, delta‐tocotrienol was the most bioactive form which was remarkably nontoxic to nontransformed pancreatic cancer cells. Both gamma and delta‐tocotrienol forms inhibited the nuclear DNA binding activity of nuclear factor kappa beta/protein 65 (NF‐kB/p65) and the delta form was more apoptotic towards pancreatic cancer compared to gemcitabine treatment.^81^ There are two other clinical trials which are ongoing; nonsmall cell lung carcinoma being treated with delta‐tocotrienol and standard chemotherapy^82^ and ovarian carcinoma, given tocotrienol synergized with bevacizumab^74^ which will elucidate the possibility of augmenting cancer treatment concomitant with tocotrienol administration.

**TABLE 1:** The latest studies on tocotrienol in cancer (published in 2021)

Model	Disease	Tocotrienol treatment regimen	Results	Outcome/Conclusion	References
Balb/c mouse	Breast cancer	Delta‐tocotrienol and its metabolite delta‐tocotrienol, 13′‐carboxychromanol in the ratio of 8/1 given mixed with diet. Duration not stated.	Reduced tumor multiplicity, reduced pro‐inflammatory cytokine release, modulation of gut microbiota	Anti‐cancer activity is promoted.	98
Human CNE1 cell line	Nasopharyngeal carcinoma	In vitro assay with delta‐tocotrienol 10–80 μm concentrations for 72 h.	Induced cell cycle arrest and apoptosis	Anti‐cancer activity is promoted.	316
MCF‐7/Adr cell line	Breast cancer	Tocotrienol 25–50 μg for 72 h with doxorubicin	NF‐kB pathway was inhibited with suppressed mdr1 promoter activity and p‐gp efflux.	Anti‐cancer activity was confirmed.	163
MBA‐MB‐231 and MBA‐MB‐453 cell lines	Triple‐negative breast cancer	Gamma tocotrienol 0–12 μM	Reduction in proliferation, migration, and EMT, inhibition of androgen receptor	Anti‐cancer efficacy determined.	317
TRF entrapped in an oil‐in‐water emulsion	Entrapment efficiency of TRF	Homogenized at 5000 rpm for 15 min with 0.75% calcium carbonate as stabilizer	Above 95% entrapment efficiency and small droplet size	Enhanced efficiency in drug delivery established.	318
Mouse +SA and TS/A mammary tumor cells	Mammary tumors/breast cancer	Gamma tocotrienol 5 and 6 μM	Attenuation of galectin‐3 distribution, blocks histamine‐induced cell migration, active binding to glycoconjugate receptors, and extracellular matrix proteins	Anticancer activity is promoted.	319
BLM human melanoma cells and A375 stem cells	Melanoma	Delta tocotrienol (data taken from abstract)	Induces paraptosis cell death, impairs mitochondria in vemurafenib‐treated A375 stem cells	Anti‐cancer activity is promoted	120
MDA‐MB‐231 cell line	Triple‐negative breast cancer	Gamma tocotrienol 8 μM with D3 (10 nM) for 4 days	Higher apoptosis, higher necrosis, lowered invasiveness	Anti‐cancer effect confirmed	320
A549, HEP G2 cell lines	Lung and liver cancer	TRF (0.05–400 μM), caffeic acid entrapped in water‐in‐oil‐in‐water multiple emulsion, and cisplatin	Enhanced cell cycle arrest at G0/G1, enhanced apoptosis, enhanced ROS, high encapsulation, and loading efficiency	Anti‐cancer efficacy determined.	321
Panc 10.05, SW 1990, AsPC‐1, BxPC‐3 cells	Pancreatic cancer	Tocotrienols with gemcitabine	Enhanced cell cytotoxicity and higher cellular uptake of gemcitabine	Anti‐cancer efficacy confirmed	322

There are several studies performed on cancer stem cells (CSC) of prostate^83^ and pancreatic cancers^84^ and human epithelial CSC from breast,^85^ colon and cervical cancers,^55^ where the delta and gamma forms of tocotrienol were markedly effective as adjuvants. These tocotrienol forms reportedly suppressed tumor progression, invasion of tissues, and metastasis, when treated combinedly with anti‐neoplastic medication (5‐azathioprine—in prostate CSC,^86^ simvastatin—in breast CSC^85^). It was achieved by the activation of several immune, metabolic, and cell signaling pathways (by downregulating signal transducer and activator of transcription 3 (STAT3) and inhibiting the mevalonate pathway, inducing Src homology 2 domain‐containing protein tyrosine phosphatase 1 and 2 (SHP1 and SHP2) proteins and mitogen‐activated protein kinase (MAPK) pathway, activation of de novo ceramide pathway, and under hypoxia through the hypoxia‐inducible 1‐alpha (HIF‐1α) signaling and strong induction of apoptosis of CSC (through the Docosahexaenoic Acid [DHA]‐induced apoptosis).^55^ Furthermore, the anticancer efficacy of tocotrienol [gamma, delta‐tocotrienol forms, and the tocotrienol rich fraction (TRF)] was demonstrated in studies involving in vitro cell lines (prostate cancer cells—LNCaP and PC3, gastric cancer cells—SGC‐7901, melanoma cells—C32 and G361), where they displayed protection against invasion, metastasis, and tumor‐angiogenesis by stimulating the E‐cadherin and beta‐catenin pathways, downregulating mRNA expression of matrix metalloproteinase‐2 and ‐9 (MMP‐2 and MMP‐9) and stimulating the inhibition of tissue metalloproteinase 1 and 2 (TMP1 and TMP2)].^55^, ^69^

The tocotrienol‐mediated anti‐cancer effects have already been established in both in vitro studies where exogenous gamma‐tocotrienol was able to suppress growth and proliferation of cells, and in vivo experiments where it had the potential to suppress tumor metastasis and angiogenesis.^87^ Another study had demonstrated gamma‐tocotrienol‐driven downregulation of tumor angiogenesis in prostate cancer cells in vitro.^88^ This was achieved through the activation of adenosine mono phosphate kinase (AMPK)‐innervated, angiopoietin‐1 (Ang‐1), and tyrosine‐protein kinase receptor‐2 (Tie‐2 receptor) inhibition which was projected as a potential downstream target of gamma‐tocotrienol in this tumor angiogenesis pathway.^88^ The researchers had noted the inhibition of Ang‐1 gene transcription from complimentary deoxyribonucleic acid (cDNA) microarray construction as well as diminished protein secretion, with gamma‐tocotrienol treatment but not tocopherol, adding a new dimension to curtailing tumor angiogenesis in advanced prostate cancer.^88^

**FIGURE 3:**
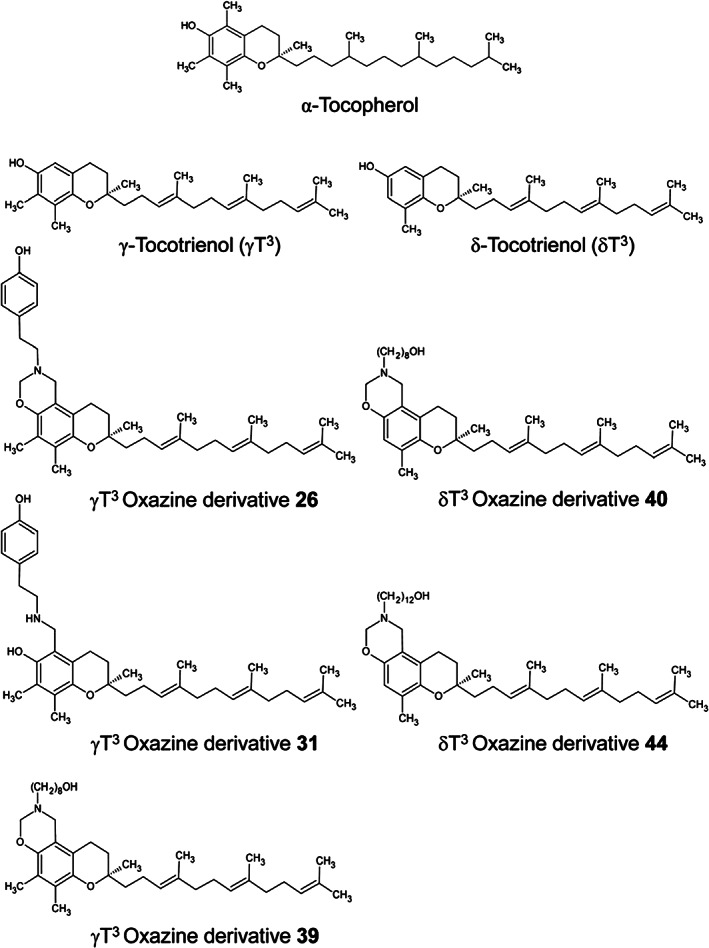
Chemical structures of gamma‐ and delta‐tocotrienol and their respective oxazine derivatives.^89^ [Reprinted from ANTICANCER RESEARCH, Vol. 34, Suryatheja Ananthula, Parash Parajuli, Fathy A. Behery, Alaadin Y. Alayoubi, Khalid A. El Sayed, Sami Nazzal and Paul W. Sylvester, “Oxazine Derivatives of γ‐ and δ‐Tocotrienol Display Enhanced Anticancer Activity In Vivo”, Pages 2715–2726, Copyright (2014), with permission from ANTICANCER RESEARCH.]

In the continued search for new therapeutics to overcome the drug‐resistance in cancers, a group of researchers had synthesized oxazine derivatives of both gamma‐ and delta‐tocotrienol as (Figure 3) a novel treatment option in in vitro and in vivo models of breast cancer.^89^ Oxazines are heterocyclic compounds that exhibit anti‐cancer effects better than the parent compounds and significantly inhibited tumor proliferation in syngeneic mouse mammary glands.^89^ The mode of action of those oxazine derivatives included suppression of Protein kinase‐B (Akt) and nuclear factor‐kappa beta (NF‐kB) pathways, increased cell cycle arrest proteins, protein 21 (p21), and protein 27 (p27) as well as the inhibition of cyclin D1, cyclin‐dependent kinase 2, 4, and 6 (CDK2, CDK4, CDK6) and cyclooxygenase‐2 (COX‐2) combined immune‐metabolic processes.^89^

The importance of gamma‐tocotrienol is further justified by its ability to influence multiple gene activation mechanisms such as cell growth, proliferation, cell cycle, cell death, cellular movement, development, apoptosis, ER stress, and gene expression itself.^90^ A study performed on breast cancer tumorigenesis using Michigan cancer foundation‐7 (MCF‐7) and M.D. Anderson‐metastatic breast 231 (MDA‐MB‐231) cell lines have revealed that gamma‐tocotrienol is a multi‐faceted anti‐cancer therapy as mentioned earlier.^90^

A simulation software linked to gene transcription analysis has revealed a significant increase in activating transcription factor‐3 (ATF3), synergized by gamma‐tocotrienol treatment. When ATF3 activity was inhibited by small interfering ribonucleic acid (siRNA) activity, gamma‐tocotrienol was able to restore ATF3‐induced apoptotic effects which in turn downregulated tumor proliferation.^91^

Radiation therapy is one of the most common adjuvant cancer therapies initiated after surgery and gamma‐tocotrienol have demonstrated significant recovery in some mouse studies such as CD2FI mouse exposed to total body gamma irradiation‐induced cell death.^92^ The radioprotective properties elicited by gamma and delta‐tocotrienol were documented to include recovery from radiation‐induced cell death via extracellular signal‐regulated kinase (ERK)/mammalian target of rapamycin (mTOR) pathways with enhanced survival and protection of hematopoietic cells, where the hematopoietic system is regarded mostly at risk from irradiation. Further, radioprotection from gamma‐ and delta‐tocotrienol forms also included cancer stem cells and progenitor cell foci in mouse bone marrow, where gamma‐tocotrienol induced and promoted hematopoiesis as well.^93^, ^94^ Additionally, gamma‐tocotrienol treatment yielded protection from radiation therapy‐induced damage in healthy cells as well as promoted irradiation‐induced cell death in tumors by increasing tumor cell sensitization to irradiation.^95^ Gamma‐tocotrienol is known to inhibit cancer cell invasion and metastasis by reversing epithelial‐to‐mesenchymal transition (EMT—being the salient mechanism of invasion and cancer metastasis), in studies involving melanoma and prostate cancer cells.^96^ Thus, gamma‐tocotrienol treatment promoted the expression of epithelial markers such as E‐cadherin and gamma‐catenin, while mesenchymal markers, alpha‐smooth muscle‐actin, and vimentin, were suppressed.^97^ Furthermore, the anti‐cancer stem cell properties of tocotrienol were linked to its potential for reversing EMT, that simultaneously hedged against disease recurrence.^97^

In a murine model of colitis‐associated colorectal cancer (CAC), treatment with delta‐tocotrienol and its metabolite, delta‐tocotrienol 13 carboxychromanol together, have displayed a role in modulating the gut microbiome composition but not richness.^98^ CAC was induced in mice by jointly administered azoxymethane and dextran sulfate sodium (DSS) in the diet that was followed by treatment with a delta‐tocotrienol mixture that could prevent the multiplicity of large adenomas in the colon.^98^ These observations suggest that tocotrienol's anti‐tumorigenic properties may also result from the modulation of the gut microbial flora because there was a conspicuous increase in the *Lactococcus* and the Bacteroides in the intervention group compared to the control group.^98^ The therapeutic combination inhibited tumorigenesis and suppressed proinflammatory cytokines in addition to influencing the gut microbial community. The modulation of the gut microflora by tocotrienols may present a mechanism toward attenuating tumorigenesis in the colon.^98^

### 2.1 Anti‐cancer mechanisms of tocotrienol

#### 2.1.1 Cell cycle arrest by tocotrienol

An increasing number of research studies investigating the chemotherapeutic action of tocotrienol has risen in recent years, which have confirmed a substantial effect on cell death in cancerous tumors through cell cycle arrest.^99^ Abrogation of the cell cycle is an effective gateway to controlling the uninhibited cell growth and proliferation observed in all types of cancer.^100^ Therefore, the regulation of cell cycle checkpoints offers a viable strategy in cancer therapy, which can be achieved by tocotrienol treatment, either alone or combined with standard chemotherapeutics.^101^ Cell cycle block has been reported with simultaneous gamma‐tocotrienol treatment in suppressing tumor growth and development in estrogen‐dependent breast cancer cells (MCF‐7) and estrogen‐independent breast cancer cells (MDA‐MB‐231),^91^ delta‐tocotrienol in hepatocellular carcinoma; HepG2 cells,^102^ gamma‐tocotrienol in prostate cancer; LNCaP, DU145, PC‐3, and human VCaP cells,^69^ delta‐tocotrienol in melanoma cells; B16,^103^ gamma‐tocotrienol in human cervical carcinoma; HeLa cells,^104^ through immune‐metabolic modulation including the upregulation of p21 and p27, and the diminishing of cyclin D1, CDK‐2, 4, and 6, and phosphorylated Rb.^105^ Tocotrienol activity in the modulation of cell cycle proteins have been reported in glioblastoma and leukemia (by delta‐tocotrienol), pancreatic cancer (by gamma‐tocotrienol), and gastric cancers (by gamma‐tocotrienol).^104^, ^106^ The suppression of brain cancer cells (U87MG) has been documented with gamma‐tocotrienol administered together with Jerantinine A, an indole alkaloid which is a potent anti‐proliferative agent that disrupted the G0/G1 interphase and microtubular polymerization, through Fas Ligand (FasL) and protein 53 (p53)‐stimulated apoptosis via the intrinsic mitochondrial pathway.^72^

Gamma‐tocotrienol combined with Doxorubicin reversed multi‐drug resistance in breast cancer in the multi‐drug resistant breast cancer cell line (MCF‐7/Adr breast cancer cells) through enhanced P‐glycoprotein expression which led to G2/M arrest and apoptosis.^107^ P glycoprotein is an ATP‐dependent drug efflux pump present in tumors. Gamma‐tocotrienol was able to arrest the cell cycle at G0/G1 phase in HeLa cells as well.^63^

#### 2.1.2 Apoptosis by tocotrienol

**FIGURE 4:**
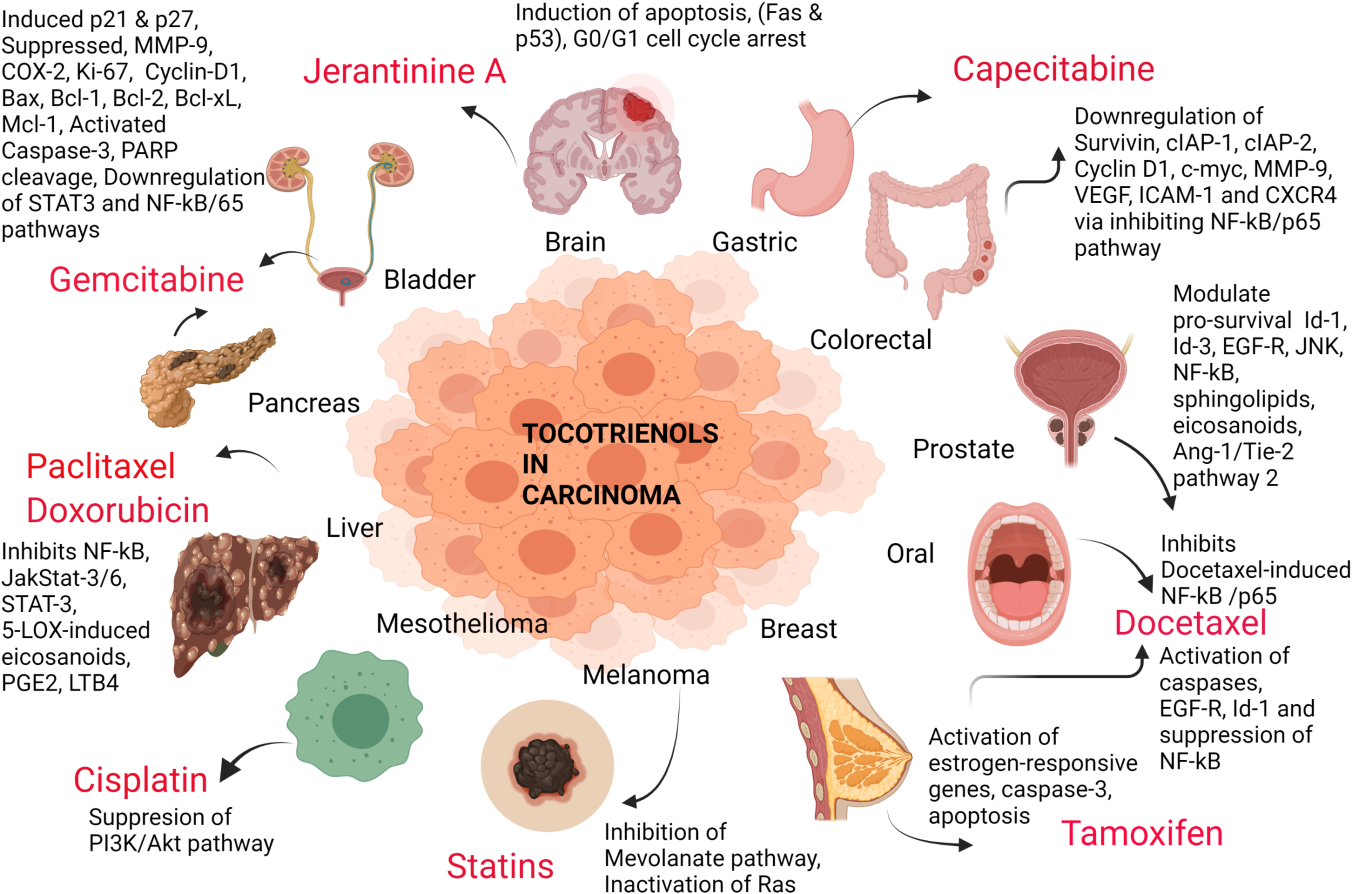
The different anti‐cancer drugs and the molecular mechanisms with which Tocotrienols have been combined successfully. p21—a cyclin‐dependent kinase inhibitor—cell cycle inhibitor in G1/S phases; p27‐another cyclin‐dependent kinase inhibitor‐regulating the cell cycle in G0 to S phases; MMP—matrix metalloproteinases—degrades matrix and non‐matrix proteins; COX—cyclooxygenase—Produces prostaglandins‐initiate inflammation; KI‐67—a nuclear protein inducing cell proliferation; cyclin D1—a cell cycle regulatory subunit of cyclin‐dependent kinases; Bax—Bcl‐2‐associated X protein—an apoptosis regulator; Bcl‐1, Bcl‐2, Bcl‐XL—B cell lymphoma family members—regulator proteins of apoptosis, cell death; Mcl‐1‐myeloid cell leukemia 1—a regulator of mitochondrial homeostasis and an apoptotic member of the Bcl—family of proteins; Caspase‐3—an apoptosis regulator in DNA fragmenting and degradation of cytoskeletal proteins; PARP—poly adenosine di phosphate (ADP) ribose polymerase—a protein which helps cells to repair by itself; STAT3—signal transducer and activator of transcription‐3—protein involved in cell growth, proliferation, migration, and apoptosis; NF‐kB/69—nuclear factor kappa beta subunit of the protein 65—induces inflammation and its progression; Jak/STAT‐3/6—janus kinase/signal transducer and activator of transcription proteins—induce cellular senescence; 5‐LOX‐induced eicosanoids—5‐lipoxygenase‐induced eicosanoids—induces prostaglandins and leukotrienes; PGE2—prostaglandin 2—inducers of inflammation; LTB4—leukotriene B4‐involved in inflammation; PI3K—phosphatidylinositol‐3‐kinase—plasma membrane associated lipid kinases involved in cell proliferation and survival; Akt—protein kinase B—involved in cell growth, proliferation, angiogenesis, vasorelaxation; Ras—a family of proteins involved in cellular functions and survival; EGF‐R—epidermal growth factor‐receptor—controls cell division and survival; Id‐1—inhibitor of differentiation‐1 associated with docetaxel—promotes longer relapse‐free survival in cancer patients; Ang‐1/Tie‐2—angiopoietin‐1 and receptor tyrosine kinase—promotes endothelial cell survival, vascular protective effects; Survivin—inhibitor of apoptosis; cIAP‐1 and 2—Cellular inhibitor of apoptosis proteins—inhibits apoptosis; c‐Myc—human oncogene overexpressed in various cancers; VEGF—vascular endothelial growth factor; ICAM‐1—intercellular adhesion molecule‐1—Facilitates leukocyte‐endothelial trans migration; CXCR4—chemokine receptor protein which spans the outer membrane of cells in white blood cells and many other cell types

**FIGURE 5:**
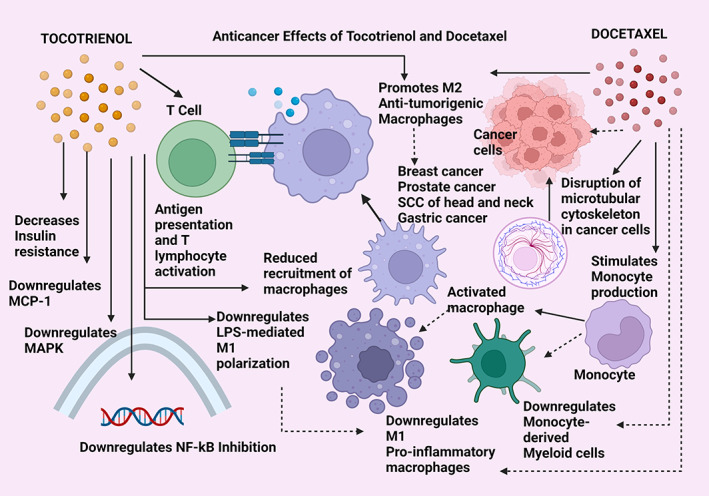
Anticancer effects of tocotrienol compared with docetaxel. MCP‐1—monocyte chemo‐attractant protein‐1; MAPK—mitogen‐activated protein kinase pathway; NF‐kB—nuclear factor kappa beta pathway; LPS—lipopolysaccharide; M1 and M2—the two broad divisions of macrophage types; SCC—squamous cell carcinoma. Straight arrows indicate stimulation/upregulation and dotted arrows represent downregulation/inhibition mechanisms.

**FIGURE 6:**
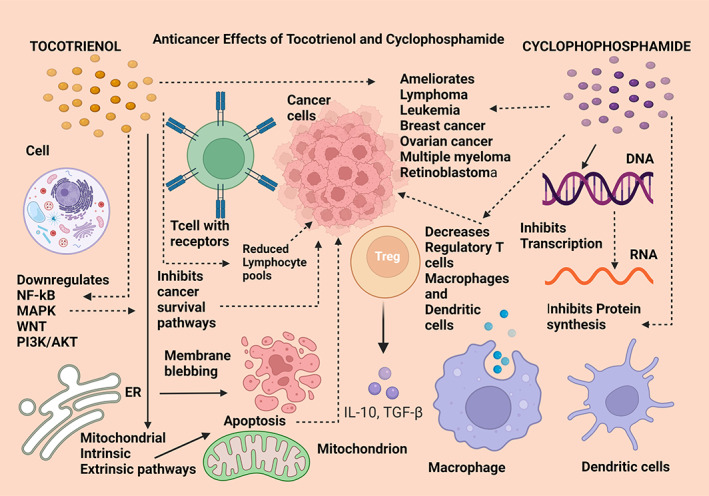
Anticancer effects of tocotrienol compared with cyclophosphamide. NF‐kB—nuclear factor kappa beta; MAPK—mitogen‐activated protein kinase; PI3K/AKT—phosphatidylinositol‐3‐kinase/protein kinase B; ER—endoplasmic reticulum; IL‐10—interleukin 10; TGF‐β—transforming growth factor‐beta; DNA—deoxyribonucleic acid; RNA—ribonucleic acid. Straight arrows indicate stimulation, dotted arrows indicate inhibition

**FIGURE 7:**
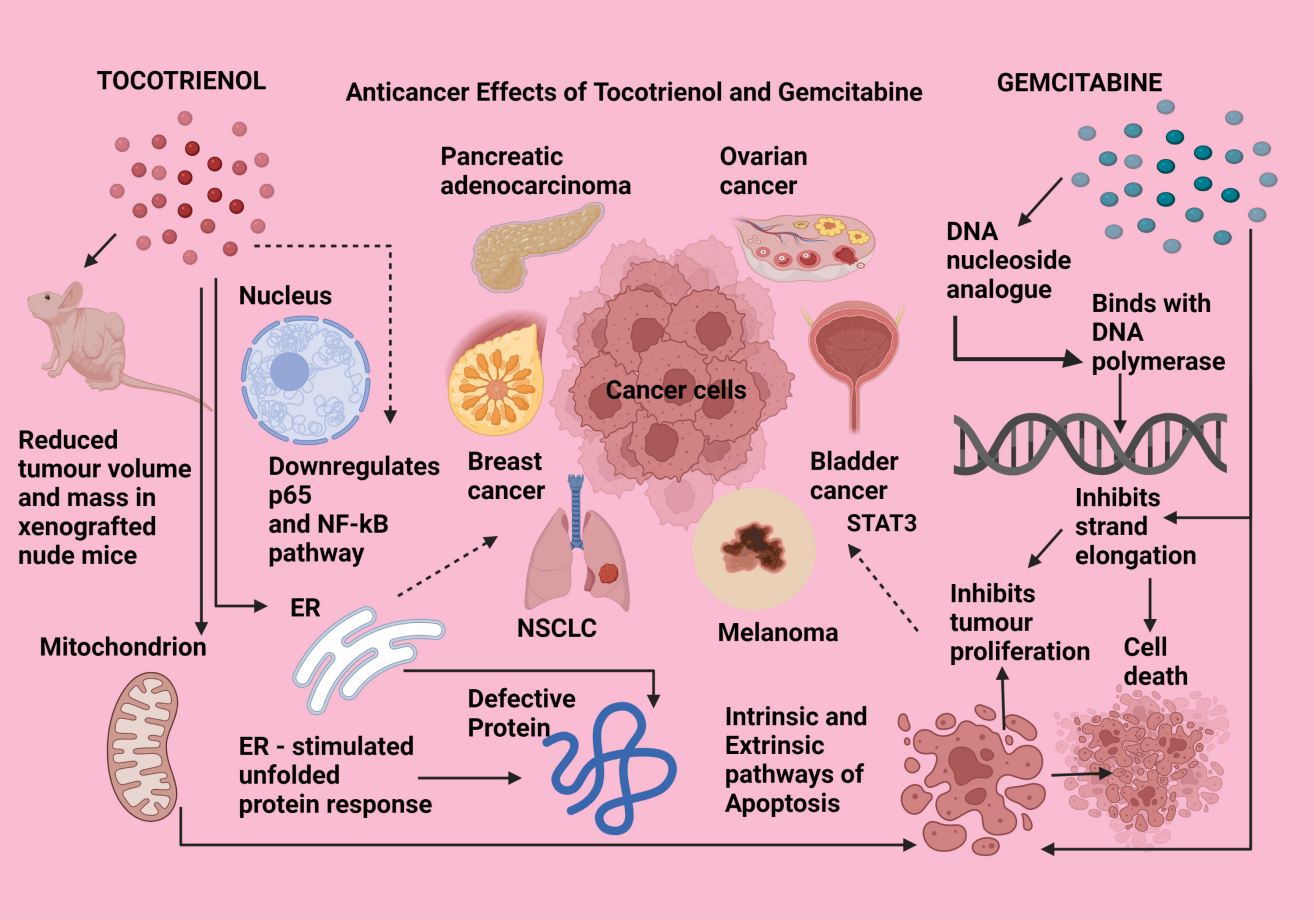
Anticancer effects of tocotrienol compared with gemcitabine. p65—protein 65; NF‐kB—nuclear factor kappa beta pathway; ER—endoplasmic reticulum; NSCLC—nonsmall cell lung cancer; DNA—deoxyribonucleic acid. Straight arrows indicate stimulation, dotted arrows indicate inhibition

**FIGURE 8:**
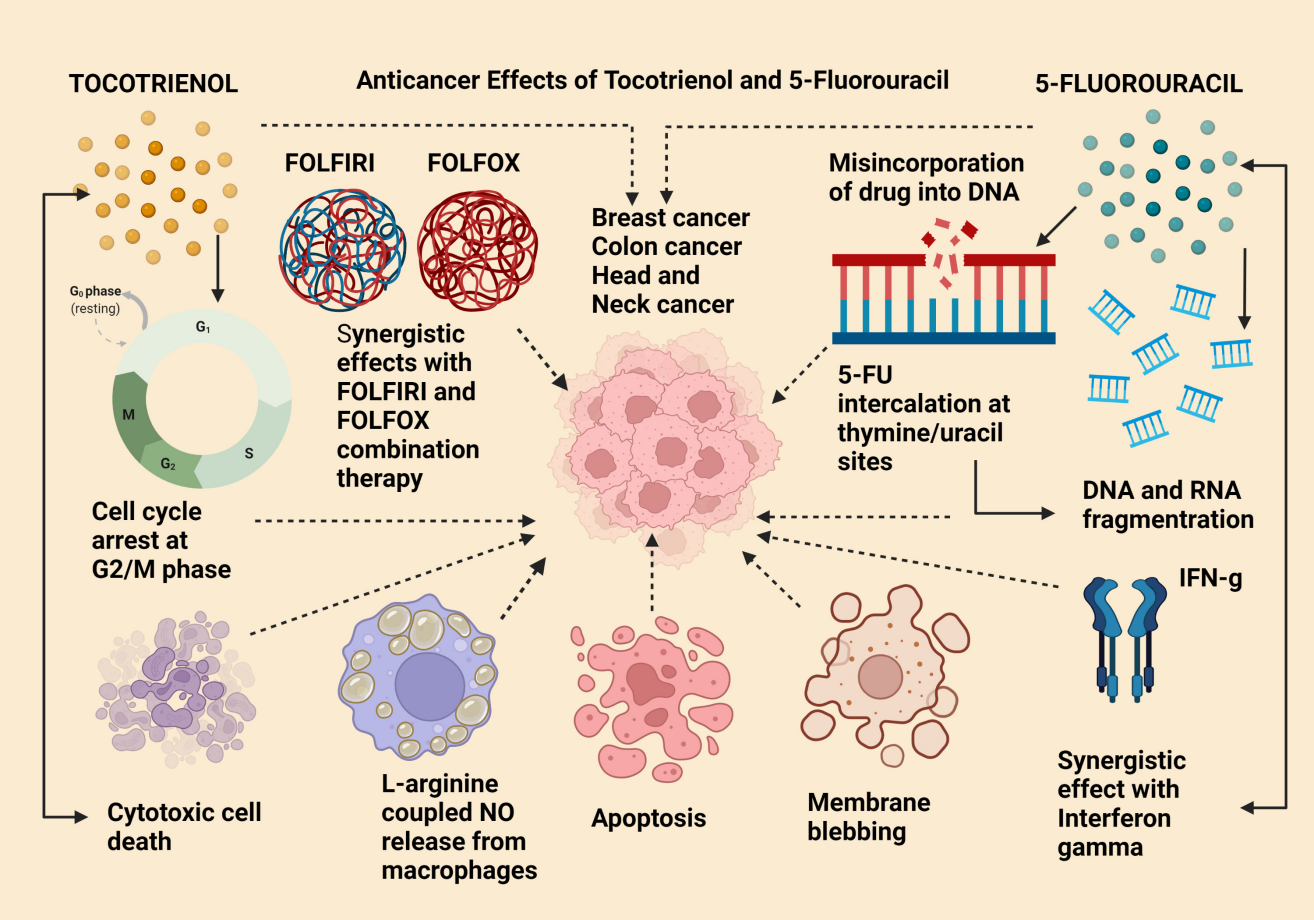
Anticancer effects of tocotrienol compared with 5‐fluorouracil. G2/M—growth phase 2/mitotic phase; NO—nitric oxide; DNA—deoxyribonucleic acid; 5‐FU—5‐fluorouracil; RNA—ribonucleic acid; IFN‐g—gamma interferon; FOLFIRI—folinic acid, leucovorin, 5‐fluorouracil, and irinotecan; FOLFOX—folinic acid, fluorouracil, and oxaliplatin. Straight arrows indicate stimulation, dotted arrows indicate inhibition

**FIGURE 9:**
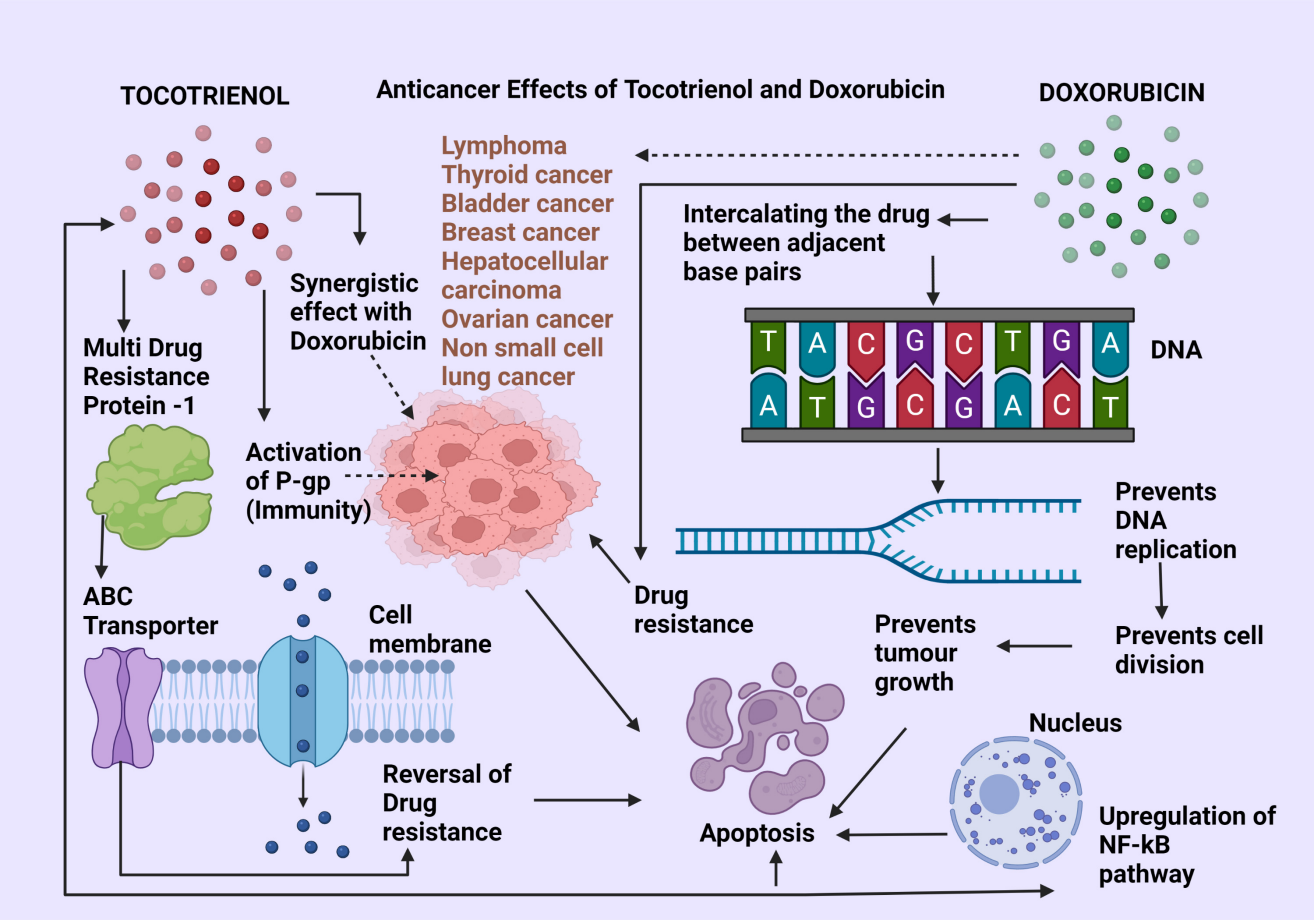
Anticancer effects of tocotrienol compared with doxorubicin. ABC transporters—ATP‐binding cassette transporters; P‐gp—protein glycoprotein; NF‐kB—nuclear factor kappa beta pathway; DNA—deoxyribonucleic acid. Straight arrows indicate stimulation, dotted arrows indicate inhibition

**FIGURE 10:**
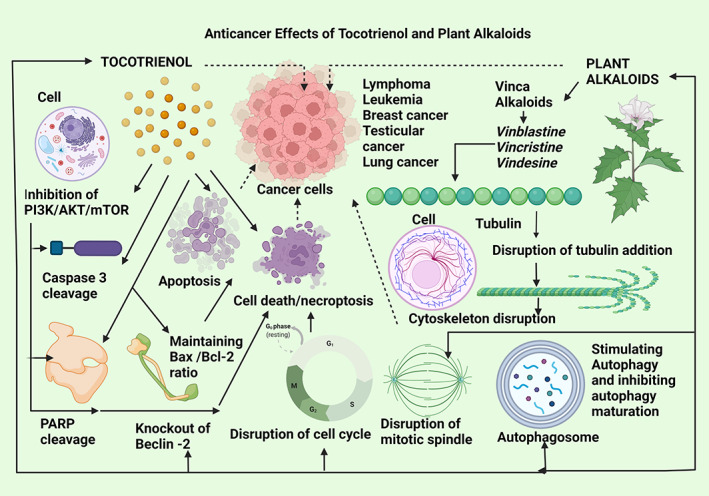
Anticancer effects of tocotrienol compared with plant alkaloids. PI3K/AKT/mTOR—phosphatidylinositol‐3‐kinase/protein kinase B/mammalian target of rapamycin, poly adenosine diphosphate‐ribose polymerase. Straight arrows indicate stimulation, dotted arrows indicate inhibition

**FIGURE 11:**
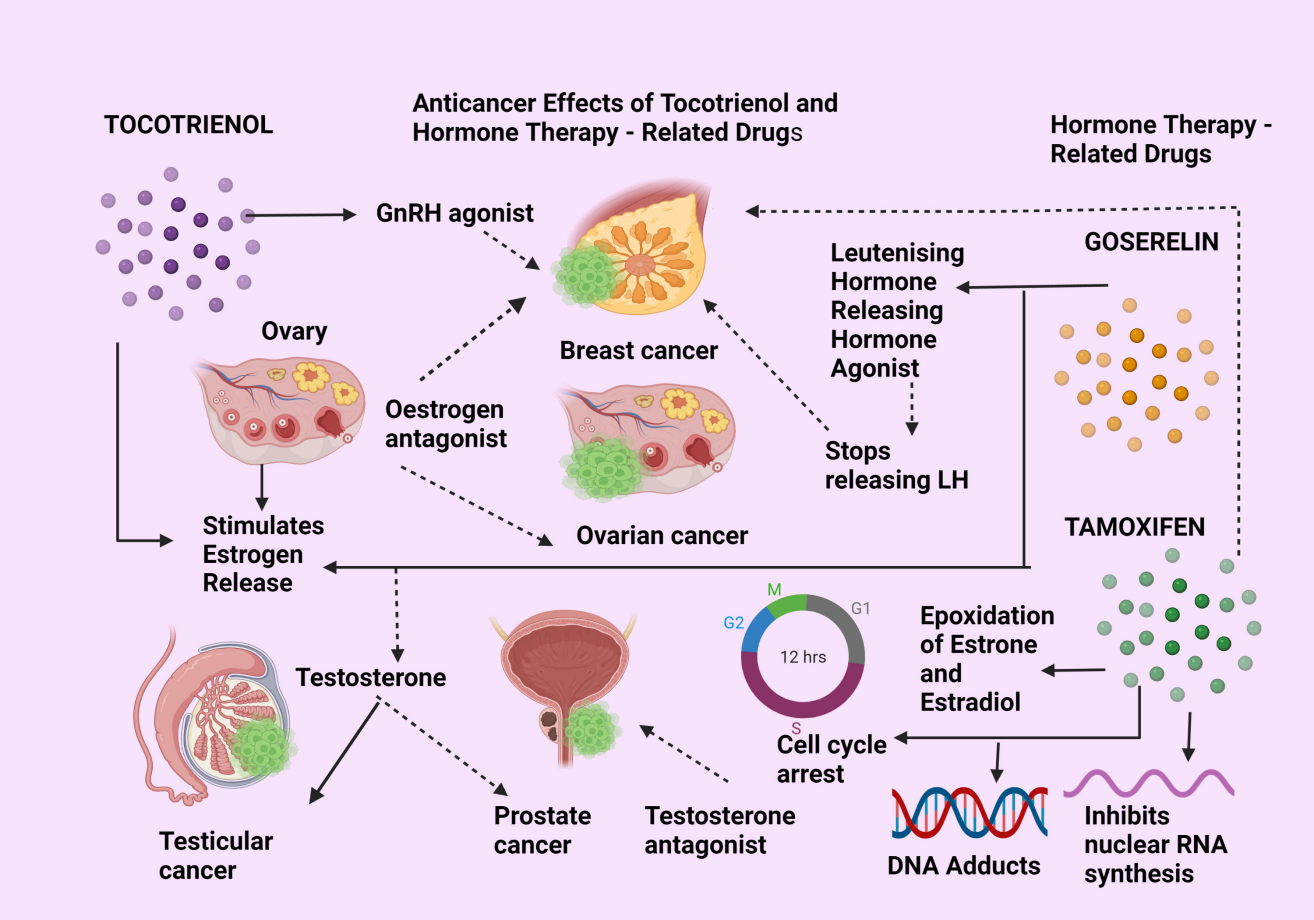
Anticancer effects of tocotrienol compared with hormone therapy. GnRH—gonadotropin‐releasing hormone; LH—luteinizing hormone; DNA—deoxyribonucleic acid; RNA—ribonucleic acid. Straight arrows indicate stimulation, dotted arrows indicate inhibition

**FIGURE 12:**
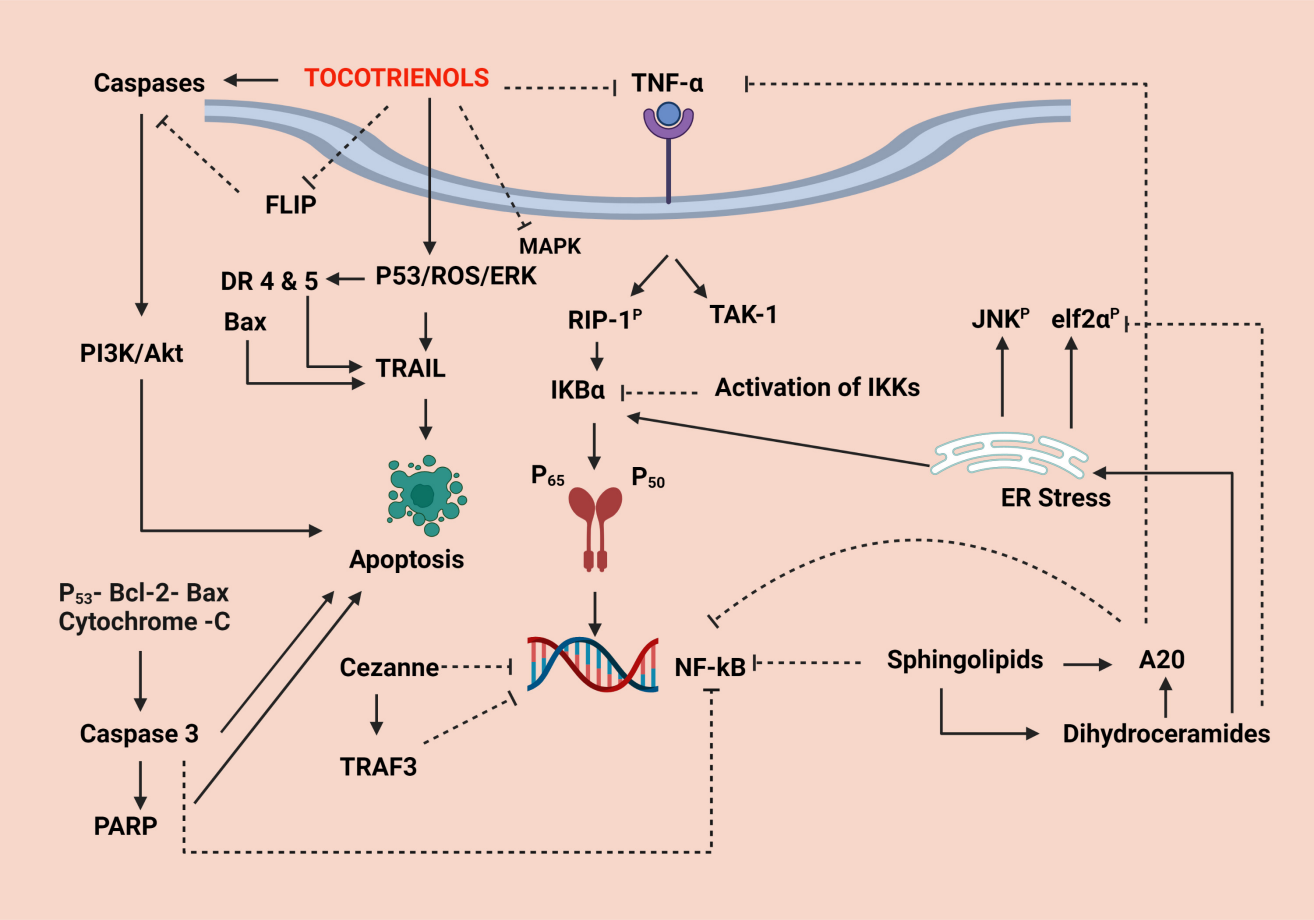
The common molecular mechanisms induced by tocotrienol therapy in experimental studies: Straight arrows indicate upregulation, and the dotted arrows show inhibition. TNF‐α—tumor necrosis factor‐alpha—a pro‐inflammatory cytokine—an inflammatory marker; FLIP—FLICE inhibitory protein—an inhibitor of apoptosis—a cytoplasmic protein complex which activates the inhibition of caspases; DR 4 & 5—death receptor 4 and 5; P‐53—A protein which acts as a tumor suppressor—inhibits uncontrolled cell proliferation; ROS—reactive oxygen species; ERK—extracellular signal‐regulated kinase; Bax—a member of the Bcl‐2 gene family which are nuclear encoded proteins which mediate apoptosis through mitochondrial activation; TRAIL—TNF‐related apoptosis‐inducing ligand—a protein ligand that induces cell death; PI3K—phosphoinositide 3—kinase, a protein which favors cellular functions in cancer; Akt—protein kinase (B)—a serine/threonine specific protein kinase—an enzyme which plays a key role in many cellular functions; NF‐kB—nuclear factor kappa beta‐pathway; PARP—poly‐ADP ribose polymerase—enables repair of DNA damage; Cezanne—deubiquitinating protein which inhibits NF‐kB translocation; TRAF3—a member of the TNF‐receptor associated factor protein family—which negatively regulates NF‐kB activity; RIP1^P^—receptor interacting serine/threonine‐protein kinase 1—Induces apoptosis and necroptosis by participating in NF‐kB, Akt, and JNK pathways; TAK1—transforming growth factor‐beta‐activated kinase‐1—mediates NF‐kB, p38, and JNK pathways, JNK^P^‐phosphorylated c‐Jun‐N—terminal kinase—controls apoptosis and cell proliferation—A member of the MAPK—mitogen‐activated protein kinase pathway; IKBα, nuclear factor of kappa light polypeptide gene enhancer in B cells inhibitor‐alpha—inhibits NF‐kB transcription; IKK—Inhibitor of upstream IKB kinase—inhibits IKB phosphorylation, degradation, subsequent NF‐kB nuclear translocation; p65—a subunit of NF‐kB nuclear transcription‐involved in inducing the NF‐kB pathway‐immunity, tumorigenesis, inflammation; p50—a component transcription factor of the NF‐kB pathway; ER—endoplasmic reticulum; elf2α^P^—phosphorylated eukaryotic translation initiation factor‐alpha‐subunit 1‐involved in translation in protein synthesis; A20—plays an anti‐tumorigenesis role in cancer

**FIGURE 13:**
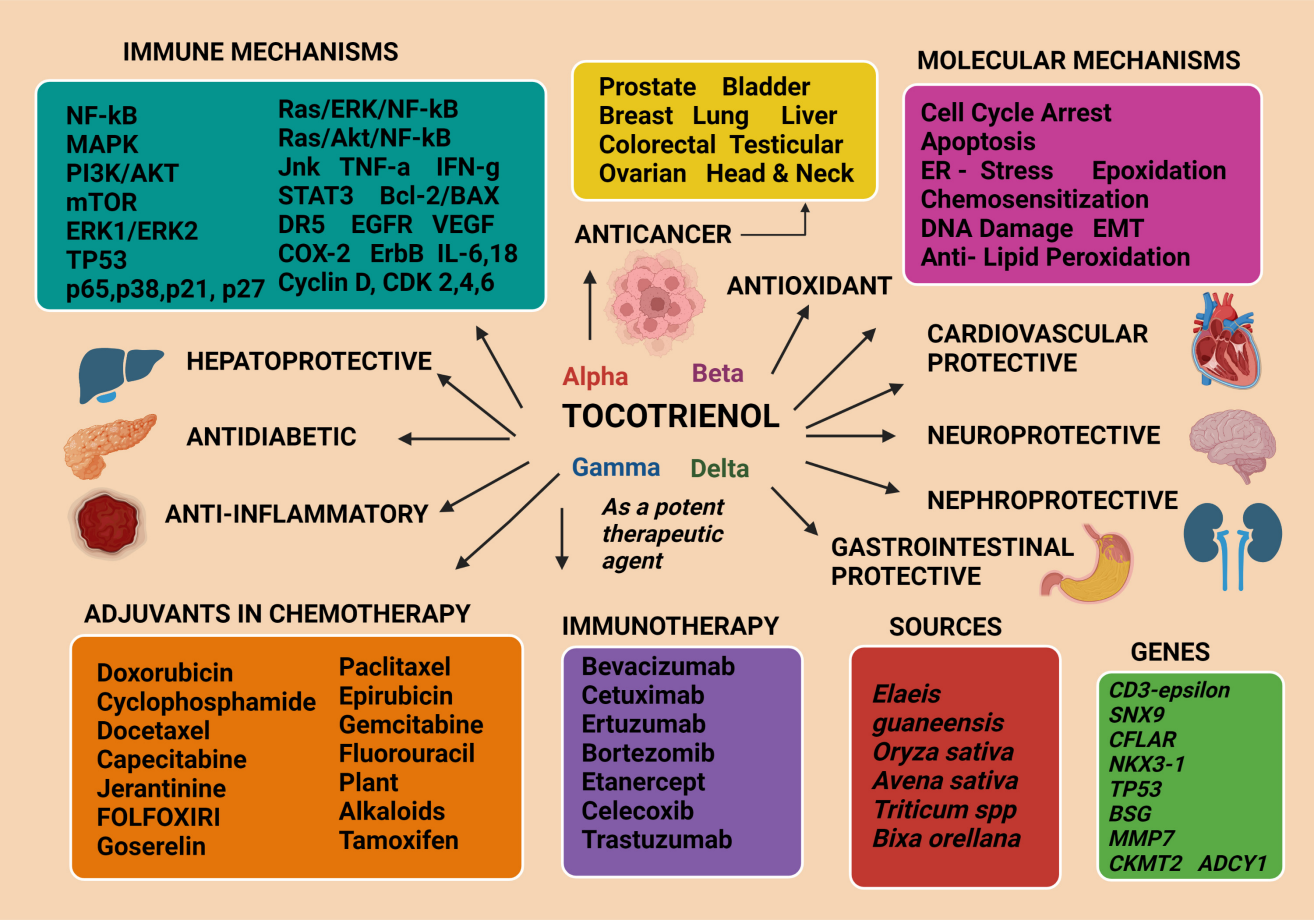
The summary of tocotrienol functions that highlight its therapeutic potential

Apoptosis is an innate immune mechanism involved in cancer cell death which is targeted by most cancer drugs. There are evidence‐based studies carried out on tocotrienol effectiveness in triggering cellular apoptosis, as a means of enhancing its anti‐tumorigenic properties.^108^ Gamma‐tocotrienol has shown potent apoptotic activity and growth inhibitory effects in prostate cancer (PCa) cells and in downregulating the expression of several oncogenic products through the inhibition of NF‐kB pathway and apoptosis, as well as anti‐invasive effects and chemo sensitization (in prostate cancer).^56^ Furthermore, gamma‐tocotrienol has demonstrated regulatory effects on B‐cell lymphoma 2 (Bcl‐2) protein and caspase‐3 activity in a gastric cancer cell line (SCG 7901), by mediating apoptosis through the rapidly accelerated fibrosarcoma–extracellular signal‐regulated kinases (Raf–ERK) signaling cascade.^109^ Additionally, studies of alpha‐tocotrienol treatment on cancerous cell lines have produced favorable results such as anti‐proliferation effects and apoptosis in human cervical cancer‐HeLa cells by the suppression of cyclin D, protein 16 (p16), CDK6, and inducing interleukin‐6 (IL‐6) expression and the mitochondrial apoptosis pathway.^100^ Triple‐negative breast cancer (MDA‐MB 231) was attenuated by activating micro ribonucleic acid (miR)‐429 and estrogen‐dependent MCF‐7 cells^110^ and inducing DNA damage, NF‐kB inhibition, and poly ADP‐ribose polymerase (PARP) cleavage.^111^ Similarly, apoptosis was induced in a breast cancer cell line (66‐cl‐4‐GFP) by the expression of death receptor 5 (DR5) dependent on c‐Jun N‐terminal kinase (JNK), protein 38 (p38), mitogen‐activated protein kinase (MAPK) pathway, and ERK‐mediated endoplasmic reticulum (ER) stress pathway.^112^ Apoptosis was induced in bladder cancer (T24, 5637, J82, and UMUC‐3) by inhibiting the signal transducer and activator of the transcription 3 (STAT3) pathway.^113^ Chronic myeloid leukemia (K562 cells) was suppressed by both intrinsic and extrinsic mechanisms,^114^ and nonsmall cell lung cancer (NSCLC) was inhibited by the downregulation of NF‐kB and Notch‐1 pathways.^115^ Gamma‐tocotrienol combined with lovastatin had induced apoptosis in HL60 cells by suppressing rat sarcoma‐derived guanine nucleotide‐binding protein, Ras/ERK/NF‐kB, and Ras/Akt/NF‐kB mechanisms and downregulating glyoxalase 1 and 3‐hydroxy 3‐methyl glutaryl coenzyme A (HMG‐CoA) reductase activity.^116^ There are more studies on which tocotrienol treatment had been fruitful, that were performed with cell lines that included human lung adenocarcinoma (A549),^117^ and glioblastoma (U87MG)^117^ by beta‐tocotrienol, cervical cancer (Caski cells),^118^ and hepatic cancer by gamma‐tocotrienol.^119^ All these studies have highlighted the potent apoptotic and anti‐neoplastic strategies possible with tocotrienol therapy that are based on its multiple effects on different aspects of immune function (Figures 4, 5, 6, 7, 8, 9, 10, 11, 12, 13). Therefore, further studies on tocotrienols could yield significant data to confirm its anti‐tumor therapeutic potential.

A study in 2021 has described delta‐tocotrienol therapy‐activated cellular paraptosis, which is noncanonical programmed cell death. Paraptosis is a prominent anti‐cancer strategy utilized to suppress the progression of melanoma.^120^ Delta‐tocotrienol was capable of inducing membrane dysfunction in both mitochondria and ER at a concentration of 15 μg/ml kept for 12 h in A375 and BLM cell lines. This treatment resulted in an overproduction and influx of calcium ions, which led to a marked increase in reactive oxygen species (ROS).^120^ Membrane dysfunction was achieved by cytoplasmic vacuolization produced by ER and mitochondrial membrane defects that caused downregulation of metabolic oxidative phosphorylation. It also induced hypoxia that diminished adenosine triphosphate (ATP) production leading to defective adenosine mono phosphate activated‐protein kinase (AMPK) phosphorylation, which are integral steps in cellular energy homeostasis.^120^ Another recent cancer research study (in 2019) has confirmed the effectiveness of delta‐tocotrienol treatment as a novel strategy in suppressing pancreatic carcinoma, because of its ability to induce apoptosis via the TNF‐alpha‐related apoptosis inducing‐ligand (TRAIL).^121^ TRAIL‐induced apoptosis which happens through the mediation of death receptors synergistically acting with caspase 8, was implicated in this study for mediating the downregulation of Fas‐associated death domain‐Like IL‐1‐converting enzyme (FLICE)‐inhibitory proteins, FLIP‐c in particular, that is known to suppress apoptosis through the action of caspase 3 cleaving enzymes.^121^ In brief, this programmed cellular apoptosis was induced by delta, gamma, and alpha‐tocotrienol forms through promoting FLIP‐c ubiquitination leading to the degradation of the FLICE‐inhibitory proteins.^121^ There are several more studies which have been published recently in 2021 (Table 1).

#### 2.1.3 Chemo‐sensitization by tocotrienol

Tocotrienols have displayed the potential to promote chemo sensitization in cancer cells, a process which increases the sensitivity of tumors to chemotherapy while reducing its toxicity in normal cells.^122^ Chemosensitivity to cytotoxic cancer treatment is recognized as a way to lower side effects arising from, present‐day anti‐cancer drugs.^122^ The ability of both gamma‐^71^, ^123^ and delta‐tocotrienol^113^ to induce chemosensitization, when treated simultaneously with a broad range of drugs, are documented in cancer‐related studies. They include the concurrent supplementation of tocotrienols with the following drugs: gemcitabine in pancreatic^60^ and bladder^113^ cancer, capecitabine in colorectal^124^ and gastric^71^ cancer, tamoxifen^125^ and docetaxel^126^ in breast cancer, paclitaxel^123^ and doxorubicin^127^ in hepatic cancer, docetaxel^128^ in prostate cancer, cisplatin^129^ in mesothelioma H28 cells, statins^130^ in melanoma cells, and docetaxel in human oral cancer cells^75^ (Figure 4).

The advantage of combination therapy is that it can reduce drug resistance in the targeted cells, improve potency, and minimize the nonselective toxicity in noncancerous cells.^72^ Stand‐alone gamma‐tocotrienol therapy in brain cancer cells had been toxic at high doses (3.17 μg/ml) and therefore, was restrictive in its therapeutic potential when used by itself.^131^ This was shown in an in vitro study on brain cancer cells which had gamma‐tocotrienol (at 1.29 μg/ml) as an adjuvant with the phytochemical, Jerantinine A (at 0.16 μg/ml), in combined therapy that was successful in promoting anti‐cancer efficacy at a lower dose.^72^ Compared to the gamma‐tocotrienol high dose, the percentage decrease in concentration at which the combined therapy was effective was 59% less than either gamma‐tocotrienol or Jerantinine given alone because Jerantinine was also highly toxic to healthy cells at 0.62 μg/ml. Therefore, the combined treatment had achieved anti‐cancerous benefits (decreased tumorigenic proliferation) through activating the Fas‐p53 signal transduction to induce apoptosis via the mitochondrial pathway.^72^ Another study on a statistical modeling system using a software called mixture design response surface methodology (MDRSM) aimed at identifying the best combination of a curative drug design for prostate cancer, has been reported.^132^ The best combination treatment was identified as gamma‐tocotrienol with alpha‐tocopherol ether acetate and docetaxel that could evade drug resistance in prostate cancer treatment than docetaxel given alone.^132^ The synergistic action of gamma‐tocotrienol and polysaccharopeptide (PSP) has been investigated in which, prostate cancer cell growth, proliferation, and cytotoxicity were seen to reduce, through the mediation of AMPK.^133^ The researchers of this study had observed an increase in AMPK with the simultaneous inactivation of acetyl‐co A carboxylase, an integral step in cellular energy homeostasis, evidenced by the Ser 79‐activated phosphorylation.^133^ Further, gamma‐tocotrienol possessed the ability to sensitize the prostate cancer tumors to PSP, in vivo.^133^

#### 2.1.4 Anti‐metastasis mechanisms of tocotrienol

Cancer metastasis is a dynamic process that underpins the need for effective cancer medicines that can potentially prevent the motility of extracellular matrix and cells within the tumors.^104^ Tocotrienols are endowed with anti‐cancer metastasis properties which could modulate the numerous extrinsic and intrinsic factors that are responsible for tumor metastasis and therapeutic resistance in most cancers. Delta‐tocotrienol exhibited anti‐metastasis properties by down‐regulating Notch‐1 and NF‐kB signaling pathways through the repression of protease activity of MMP‐9/urokinase‐type plasminogen activator (uPA) in NSCLC.^134^ Delta‐tocotrienol has further produced anti‐metastasis ability in pancreatic tumors by attenuating tumor cell migration, invasion, and EMT, involving vimentin, E‐cadherin, and N‐cadherin transition.^81^ Gamma‐tocotrienol was effective in repressing metastasis in in vitro breast carcinoma by the suppression of cytoskeletal re‐organization and cellular membrane protrusions. Gamma‐tocotrienol further demonstrated anti‐metastasis effects in breast tissue by inhibiting EMT through the canonical Wnt signaling mechanism.^97^ Interestingly, most of the in vitro studies that were conducted were corroborated with in vivo research that has validated the anti‐metastatic effects of tocotrienols in malignant tumors. A plethora of such studies are on record and a handful is mentioned here, that include cell lines from colorectal, prostate, cervical, melanoma, neuroblastoma, and ovarian cancers.^104^

### 2.2 Similarities between anti‐cancer mechanisms of tocotrienol and common chemotherapeutics

The existing anticancer chemotherapies belong to five broad classes: alkylating agents, anti‐metabolites, anthracyclines, plant alkaloids, and hormone‐regulated anticancer drugs. This section summarizes the similarities between these different chemotherapeutic groups and tocotrienol in cancer therapy. The aim is to draw a parallel with the chemotherapeutic molecular mechanisms that are compatible or synonymous with the tocotrienol therapeutic potential. Given the similarities which are described in the subsections below, a more detailed study to match the pharmacokinetics of tocotrienols and toxicity profile with those drugs in preclinical studies is warranted. The hypothesis is that the immune and molecular anti‐cancer mechanisms between the two groups are not dissimilar and therefore tocotrienol possesses chemotherapeutic properties that can be utilized in the treatment of cancers.

#### 2.2.1 Tocotrienol and docetaxel

Docetaxel is one of the standard chemotherapies used particularly against metastatic breast and prostate cancer and its primary mechanism of action is promoting the disruption of the microtubular cytoskeleton in cancer cells. Its main immune effect is to promote the proinflammatory activation of the M1 phenotype of the tumor‐associated macrophages and the induction of antigen‐presenting cells to activate T cytotoxic lymphocytes. The polarization of macrophages into its pro and anti‐inflammatory responses is mostly mediated and determined by the tumor microenvironment. Docetaxel is a Taxane‐derivative that produces anti‐tumor immune activation involving the monocyte‐derived macrophage activation and the suppression of the NF‐kB pathway. Tocotrienols are being used as an adjuvant with Docetaxel in an ongoing clinical trial (NCT02909751) that is investigating its potential as a combined anti‐neoplastic therapy. The immune effect of gamma‐tocotrienol is evident by its ability to induce the suppression of the monocyte‐derived proinflammatory macrophages. The interesting connection between Docetaxel and gamma‐tocotrienol is the immune activation of both these therapeutics leading to the stimulation of the macrophage repertoires that mediate an anti‐inflammatory, anti‐tumorigenic effect (Figure 5).^135^, ^136^ Tocotrienols have shown increased chemo‐sensitization when administered as an adjuvant with Docetaxel, where gamma‐tocotrienol served to increase the Docetaxel‐induced apoptosis in melanoma and prostate cancer.^60^ Gamma‐tocotrienol combined with Docetaxel showed enhanced tumor‐suppressive effects in mice implanted with breast cancer than when treated by the two therapeutics separately.^60^ Synergistic anti‐cancer effects in prostate cancer were reported by co‐treatment of Docetaxel and gamma‐tocotrienol on apoptosis mechanisms by upregulating the pro‐apoptotic proteins (caspases 3, 7, 8, 9, and cleaved PARP) and downregulating pro‐survival proteins (Id‐1, NF‐kB, p65, ik‐B, and EGF‐R).^56^

#### 2.2.2 Tocotrienol and cyclophosphamide

Cyclophosphamide is an anticancer drug that is a potent immune‐suppressive agent, prescribed mainly for various leukemias, lymphomas, breast cancer, multiple myeloma, ovarian cancer, and retinoblastoma. It was shown to downregulate both humoral and cell‐mediated immune responses where its main pharmacokinetic action is selectively suppressing the regulatory T cells and being an alkylating agent, inhibiting transcription of DNA to RNA which disrupts protein synthesis. Cyclophosphamide has produced remarkable effects in reducing the lymphocyte proliferation with markedly diminished macrophage and dendritic cell populations in peritoneal exudates. Cyclophosphamide has been combined with tocotrienols (with no information on the exact form of vitamin E) as a synergistic adjuvant therapy in clinical trials (NCT02909751) which are ongoing at present and in drawing a parallel with cyclophosphamide mode of action, tocotrienols too, possess anticancer activity by reducing pools of lymphocytes as well as achieving anti‐tumor capabilities by inducing cell death. They modulate cancer cell survival via the mitochondria and ER ‐stimulated apoptosis mechanisms and by the downregulation of cancer cell survival pathways including NF‐kB, MAPK, Wnt, and phosphatidylinositol‐3‐kinase/protein kinase B (PI3K/AKT) immune regulators (Figure 6).^137^, ^138^

#### 2.2.3 Tocotrienol and gemcitabine

Gemcitabine is an anticancer drug that belongs to the antimetabolite category of cancer drugs that is very effective against pancreatic adenocarcinoma, and solid tumors in breast, bladder, ovarian, and nonsmall cell lung cancer. It is a DNA nucleoside analog that exerts multiple mechanisms of action, of which the main action is to incorporate gemcitabine into DNA polymerase that prevents strand elongation. It also stimulates apoptosis via the intrinsic and extrinsic mitochondrial cytolytic pathways as well as the ER stress‐innervated unfolded protein response. In pancreatic tumors, gemcitabine targets the knockdown of the p65 subunit in the NF‐kB pathway, thereby causing immune suppression and abrogation of cell proliferation as the combined result of the different anticancer modalities of gemcitabine. Similar to the anti‐neoplastic action of gemcitabine, tocotrienols also display similar action by suppressing malignant tumorigenesis, evidenced by the activation of intrinsic apoptosis in human melanoma cell lines BLM and A375 in vitro while leaving the normal melanocytes unaffected. The similarity of gemcitabine and delta‐tocotrienol is that they both activate intrinsic apoptosis and ER stress‐related anticancer action. Further, the A375 xenografts in nude mice treated with delta‐tocotrienol resulted in hampered tumor progression and marked reduction in tumor volume and mass, indicating the efficacy of delta‐tocotrienol as a cytolytic agent in cancers (Figure 7).^139^, ^140^ Gamma‐tocotrienol showed increased sensitivity with gemcitabine in suppressing orthotopic pancreatic tumors in both in vitro and in vivo models.^60^

#### 2.2.4 Tocotrienol and 5‐fluorouracil

5‐Fluorouracil (5‐FU) is another antimetabolite type of anticancer drug which is now outdated due to the loss of chemosensitivity. Therefore, it is used in combined therapy to attenuate breast, colon, and head, and neck cancers. Its mechanism of action is the inhibition of thymidylate synthase which prevents the incorporation of thymine nucleotides thereby interfering with DNA synthesis, replication, and finally promoting cell death. Additionally, 5‐FU causes misincorporation with DNA and RNA polynucleotides in place of thymine and/or uracil, stimulation of apoptosis, cytotoxic cell death, cell cycle arrest, synergistic effects with gamma interferon, and conjugation with l‐arginine to increase endogenous nitric oxide release from macrophages. Combination therapy with FOLFIRI and FOLFOX was also shown to be effective in prolonging the lifespan of advanced metastatic cancers. In relating these mechanisms with tocotrienol cytotoxic and cytolytic action, in SW48 and Caco‐2 colonic cancer cell lines, the combination of delta‐tocotrienol with 5‐FU had enhanced antitumorigenic effects by way of DNA fragmentation, membrane blebbing, cell cycle arrest at the G2/M phase, nuclear condensation, and increased apoptosis. It is of interest that either 5‐FU and tocotrienol single therapy or in combined therapy, both 5‐FU and delta‐tocotrienols have achieved similar outcomes in promoting anticancer mechanisms of action particularly, in colorectal cancer (Figure 8).^141^, ^142^ This means there was no additive effect or synergism when treatment was combined or given alone.

#### 2.2.5 Tocotrienol and doxorubicin

Doxorubicin belongs to the anthracycline category of anticancer drugs which interferes with both DNA and RNA synthesis by intercalating with the adjacent DNA base pairs, thus blocking DNA replication and therefore, cell division. Doxorubicin is a highly efficacious anticancer drug that is prescribed for many cancer types, including lymphomas, nonsmall cell lung cancer, ovarian, bladder, thyroid, and breast cancers. Its main mechanism is to slow down or impede cancer growth by inhibiting Topoisomerase II which is required for cancer growth. A study on a hepatocarcinoma cell line (HCC) and breast cancer treated with Doxorubicin has revealed drug resistance, which was thought to happen via the induction of the multidrug‐resistant protein‐1 (MDR‐1) belonging to the ATP‐cassette binding protein family (ABC transporters) which occur due to changes in the tumor microhabitat and its associated signaling cascades. It is of interest to note that gamma‐tocotrienol can reverse the MDR type 1 protein action in breast cancer as well as provide a synergistic effect with Doxorubicin on the HCC cell line. It exerts its effects through conjugated therapy as well as stimulating proapoptotic mechanisms to abrogate cancer cell proliferation while augmenting Doxorubicin accumulation inside the HCC cell line. The reversal of drug resistance in tumors was achieved by gamma‐tocotrienol treatment through inhibition of the NF‐kB pathway and P‐glycoprotein (P‐gp) action which controls Doxorubicin efflux through the plasma membrane‐bound receptors (Figure 9).^107^, ^143^

#### 2.2.6 Tocotrienol and plant alkaloids

Plant alkaloids are another group of anticancer agents in use as chemotherapeutic drugs and they are a highly diverse group of compounds available for cancer therapy. Around 3000 different plant alkaloids with the US FDA approval for cancer therapy exist. Among those, indole vinca alkaloids are of importance for Vinblastine, Vincristine, and Vindesine, extracted from the plant, *Catharanthus roseus* in the family Apocynaceae have been tested for anticancer efficacy in combination therapy. They were used against lymphomas, leukemias, breast, testicular and lung cancers, and Kaposi's sarcoma. The chief mechanism of action is stimulating autophagy in cancer cells and inhibiting autophagy maturation in those tumors. Vinblastine combined with an autophagy stimulator (C‐ceramide), had induced autophagy and apoptosis in cell lines from hepatocarcinoma and colon cancers. The main mechanism of action is the disruption of tubulin addition and prevention of the mitotic spindles which lead to tumor cell death while arresting the cell cycle and hence cancer cell proliferation. The knockout of Beclin‐2, a protein associated with autophagy is also suppressed by vinca alkaloid combination therapy. In comparison, gamma‐tocotrienol also possesses the capability for inducing autophagy as shown in breast cancer and prostate cancer cells in which it stimulated an increased Beclin‐2 and Bax/Bcl‐2 ratio, cleaved caspase 3, cleaved PARP, and diminished signaling of PI3K/AKT/mTOR activation that adds to anticancer properties of tocotrienol in inducing apoptosis and autophagy leading to tumor cell death (Figure 10).^144^, ^145^

#### 2.2.7 Tocotrienol and hormone therapy

Hormone therapy is another category of anticancer treatment which is essentially used against sex hormone‐related cancers, such as breast, ovarian, testicular, and prostate cancers. Anti‐neoplastic drugs, Tamoxifen, and Goserelin are examples of hormonal/endocrine anticancer therapy which blocks the gonadotropin‐associated hormone release to suppress cancer proliferation that occurs due to increased endogenous hormone release. Another effective treatment modality is using luteinizing hormone receptor agonists, which help to suppress tumor growth. Goserelin when given as an adjuvant with radiation therapy for advanced prostate cancer was successful in extending survival in those patients while Tamoxifen, an estrogen antagonist is used to treat breast cancers in premenopausal women. Tamoxifen inhibits the epoxidation and activation of the two steroid hormones, oestradiol, and oestrone, which allows for DNA adduct formation as well as inhibiting nuclear RNA synthesis, which prevents cell division, caused by G2/M phase cell cycle arrest. The therapeutic value of epoxides comes from enzyme inhibition, cell cycle arrest, and apoptosis. Goserelin functions as an agonist analog of the gonadotropin‐releasing hormone that is used for blocking testosterone release in advanced prostate cancer treatment. Interestingly, tocotrienols too have been able to act as estrogen hormone antagonists, where both estrogen receptor‐negative and positive breast cancer cell lines had shown improved results in suppressing cancer cell proliferation either as single tocotrienol isomers or combinedly as the tocotrienol‐rich fraction (TRF). There are studies in which tocotrienols have been combined with Tamoxifen to treat breast cancer, where both together had been more synergistic in their anticancer action. Further, tocotrienols were reported to stimulate the gonadotropin‐releasing hormone (GnRH) agonists in a study of bone loss induced by testosterone deficiency (Figure 11).^146^, ^147^, ^148^, ^149^

### 2.3 Tocotrienol in cancer immunology

Tocotrienol as adjuvants in immunotherapy has made promising advances in cancer treatment. A mouse model induced with a mammary tumor has revealed tocotrienol efficacy as an adjuvant in a vaccine, containing dendritic cells (DCs) important for antigen‐sampling and conveying the antigens for priming of inactive lymphocytes. Sub‐dermally injected TRF, together with dendritic cells and tumor lysate showed a better immunogenic response (with increased secretion of the pro‐inflammatory cytokines; gamma‐interferon and interleukin‐12), in attenuating tumor growth, compared to the control group given only the DCs, vehicle and the tumor lysate. The intervention group also produced more cytotoxic CD8 cells and natural killer cells in the peripheral circulation.^150^ There is more evidence on the ability of tocotrienol to enhance DC‐mediated tumor immunity where tocotrienol had facilitated tumor elimination with increased lymphocyte proliferation. Adding to these observations is another reported study in which Inhibitor of differentiation/DNA binding Id‐1 (a member of the helix–loop–helix protein family expressed in actively proliferating cells), and beta‐catenin, two stem cell proteins promoted stem cell survival and their self‐renewal. Self‐renewal is the process of perpetuating the stem cell pool throughout life and division with the maintenance of the undifferentiated state. This requires cell cycle control and often maintenance of multipotency or pluripotency, depending on the stem cell type.^60^ There are many more studies that have reported the induction of the NF‐kB^151^ and PI3K/Akt^152^ and the inhibition of MAPK^153^ signaling cascade in suppressing tumor growth, proliferation, survival, and metastasis. Delta‐tocotrienol was able to significantly inhibit the release of pro‐inflammatory cytokines—tumor necrosis factor‐alpha (TNF‐α), IFN‐γ, IL‐1β, and IL‐6, in lipopolysaccharide (LPS) induced RAW 264.7 macrophages in addition to suppressing the phosphorylation of JNK and the ERK1/2 and MAPK pathways.^154^ There are several different immune‐modulatory treatments which have been combined with tocotrienol as therapy for alleviating disease. They are celecoxib [which inhibits cyclooxygenase (COX)‐2], cetuximab [epidermal growth factor receptor (EGFR)], bortezomib (NF‐kB), etanercept (TNF‐alpha), trastuzumab [human epidermal growth factor receptor (HER)‐2], and bevacizumab [vascular endothelial growth factor (VEGF)] modulators.^108^ The monotherapy of celecoxib, an anti‐cancer medication is limited in use by severe gastrointestinal and cardiovascular toxicity. When combined with gamma‐tocotrienol, it produced more potent inhibitory effects on cell proliferation by blocking the anti‐inflammatory COX‐2‐dependent as well as independent pathways along with decreased prostaglandin (PG‐2) levels and suppressed phosphorylated Akt and NF‐kB immune mechanisms.^155^ Monotherapy of gamma‐tocotrienol is also limited by its inability to obtain and sustain effective therapeutic levels in tissues but the synergized action of celecoxib with gamma‐tocotrienol has produced enhanced anti‐cancer therapeutic effects. This effect was achieved at the concentrations of 0.25 μM gamma‐tocotrienol and 2.5 μM celecoxib in highly malignant in vitro + SA mammary epithelial cell cultures.^155^ The clinical success of cetuximab or trastuzumab, which are monoclonal antibody therapy that targets ErbB receptor‐mediated anti‐neoplastic effects, was diminished as the receptors of the ErbB family together act to rescue mammary cancer cells from anti‐proliferative agents targeting single ErbB receptors. When combined with gamma‐tocotrienol, this EGF‐mediated anti‐cancer immune therapeutic drug produced an improvement by inhibiting the ErbB3 heterodimer transphosphorylation and selectively inhibiting the interactions between the ErbB3 receptor with the other ErbB receptor family members.^156^ The VEGF‐targeted combined delta‐tocotrienol with bevacizumab therapy elicited a favorable outcome in refractory ovarian cancer.^74^ The overall survival of patients was more in which HOXA9, a circulating tumor DNA biomarker of prognostic value was increased. Its usefulness was that the ineffective treatment regimens could be stopped during the early disease stages, suggesting that tocotrienol exerts an additive effect on bevacizumab in innervating anti‐tumor angiogenesis which the researchers of this study thought was necessary to suppress cancer progression.^74^

The primary immune/metabolic mechanism associated with tocotrienol activity appears to be the inhibition of the NF‐kB pathway,^157^ which is a central transcription factor that stimulates several genes involved in cell survival, proliferation, and inflammation (all of which are cancer‐promoting cellular metabolic processes).^158^ In resting conditions, NF‐kB remains inactive, bound to the nuclear proteins p65 and p50 which are sequestered by the inhibitory nuclear factor of kappa light polypeptide gene enhancer in B‐cells inhibitor, *alpha* **[**IKBα—a 40 kDa protein that inhibits NF‐kB action].^159^ When pro‐inflammatory cytokines are released, such as TNF‐alpha, a signaling cascade commences with the stimulation of receptor proximal signaling complexes containing receptor‐interacting protein serine/threonine kinase (RIPI) and transforming growth factor‐β (TGF‐β)‐activated kinase 1 (TAK1), which begin the phosphorylation of RIPI leading to the activation of IKBα.^160^ Activated IKBα releases the p65 and p50 dimers that translocate to the nucleus and bind to the respective gene promoters.^160^ Tocotrienol exerts its effects *by* inactivating NF‐kB via sphingolipids, which in turn activate A20 (an immunogenic protein related to both innate and adaptive immunity), which can inhibit the NF‐kB activity during the early stages of transcription.^160^, ^161^ The sphingolipids activate A20, by first stimulating dihydroceramides. The biosynthesis of dihydroceramides is initiated by serine combining with palmitoyl‐coenzyme A which is catalyzed by serine palmitoyl transferase to produce 3‐ketosphinganine which is converted to sphinganine. Sphinganine produces dihydroceramide when catalyzed by dihydroceramide synthase.^162^ Dihydroceramides promote ER stress by upregulating p‐IKBα, the protein of Jun N‐terminal kinase (p‐JNK), and translation initiation factor 2 (p‐eIf2α), those of which induce the action of A20 alongside Cezanne. Cezanne is a negative regulator of the NF‐kB by deubiquitinating TRAF3, an inhibitor of NF‐kB.^160^, ^161^ A20 with or without Cezanne can induce the TNF‐alpha receptor blockade by the TAKI, JNK, and NF‐KB pathways.^161^

Gamma‐tocotrienol treatment as an adjuvant in breast cancer therapy was found to reverse the action of MDR‐1, by inhibiting the NF‐kB pathway.^163^ Drug resistance is a considerable problem encountered in cancer therapy and the P‐gp, a plasma membrane protein pump, is associated with promoting drug efflux, thus inducing suboptimal or no drug accumulation within cancer cells.^164^ The MDR phenotype is critically associated with the overexpression of ABC transporters of which the P‐gp plays a pivotal role.^165^ Gamma‐tocotrienol action in cancer treatment attenuated the expression levels of MDR‐1 and P‐gp mRNA, in an in vitro study utilizing MCF‐7/Adriamycin breast cancer cell line, which also demonstrated its ability to reverse the drug efflux by using NF‐kB agonists/antagonists, MDR‐1 promoter activity, and P‐gp transporter activity.^163^ The TRF induced apoptosis in another experiment conducted on the breast cancer cell line, MDA‐MB‐231.^111^ The results indicated the induction of PARP cleavage (a hallmark of the cellular apoptosis process) with the inhibition of the NF‐kB pathway, leading to the suppression of unlimited cell proliferation ending up in anti‐tumorigenesis action.^111^

In addition to the inhibitory modulation seen on the NF‐kB signal transduction by tocotrienol and specifically by gamma‐tocotrienol, the PI3K/Akt signaling mechanism inhibition also is reported by other studies.^166^ PI3K/Akt pathway is a mitogen‐dependent signaling cascade which leads toward marked increases in cell proliferation, thus worsening the tumorigenesis observed in cancer patients.^166^ The upside of tocotrienol is that it does not affect the normal cell viability, but suppresses PI3K/Akt signaling by activating caspases, particularly downregulating FLIP, an endogenous caspase inhibitor in mammary tumor cells.^166^

Beta‐tocotrienol was reported to be a more potent anti‐cancer therapeutic tocotrienol form than the gamma‐tocotrienol in modulating a host of metabolic pathways, in an in vitro study performed on two breast cancer cell lines.^167^ This study investigated the function of the apoptosis‐inducing proteins, p‐53, Bcl‐2, Bax, cytochrome C, cleaved PARP‐1 and caspase‐3, and key cell survival proteins; p‐PI3K and p‐GSK3‐α/β, which reported high sensitivity to the treatment and improved anti‐cancer efficacy, and hence was introduced as a more promising therapeutic agent given its innervation of multiple immune/metabolic pathways.^167^ Beta‐tocotrienol was speculated to act via a p53‐independent, PI3K/Akt signaling pathway.^167^ Additionally, the therapeutic action of beta‐tocotrienol executing a different anticancer mechanism to the previous study was published as in vitro studies on human lung and brain adenocarcinoma cells.^117^ Anti‐cancer effects were produced by inducing double‐stranded breakages in DNA leading to apoptotic effects with chromatin condensation and formation of apoptotic bodies.^117^ The underlying mechanism of beta‐tocotrienol was described as activating the cell death receptors and caspase‐8‐mediated mitochondrial apoptosis pathway.^117^

Another mechanism of gamma‐tocotrienol was to induce TRAIL in human colon cancer tumor cells, by working through the death receptors (DR4 and 5) for which p53 and Bax upregulation are also required.^168^ TRAIL is known to act as an apoptotic mechanism, which promotes anti‐cancer properties by downregulating cell survival proteins.^168^ The TRAIL response was upregulated by gamma‐tocotrienol acting via ERK1, because ERK1 sequestration by siRNA subdues TRAIL, and was concluded that TRAIL upregulation by gamma‐tocotrienol proceeds through the stimulation of ROS/ERK/p53 pathway.^168^

In prostate cancer cells (LNCaP) compared with a normal prostate cell line (PC‐3), the gamma‐tocotrienol therapeutic mechanism was identified to upregulate apoptosis by activating c‐JUN, caspases 3 and 9, and the Ras/Raf/ERK pathway.^169^ The results indicated no therapeutic effect with alpha‐tocopherol on in vitro anti‐cancer therapy compared to gamma‐tocotrienol, irrespective of androgen sensitivity in those cells.^169^ The mode of action of both gamma and delta‐tocotrienol on pancreatic cancer cell lines showed significant anti‐proliferative and apoptotic ability by suppressing; ERK/MAP kinase, phosphorylation of Akt, and ribosomal protein S6 kinase (RSK), through the downregulation of Her2/ErbB2 mechanism at the messenger level.^170^ Another contradictory study on epidermal growth factor (EGF)‐induced neoplastic +SA mammary epithelial cells reported that gamma‐tocotrienol exerts its anti‐proliferative effects by the upregulation of ErbB3 tyrosine receptor phosphorylation (with heterodimerization), and not by ErbB1 and ErbB2 receptor mediation.^156^ However, it was reported that optimal signal transduction with the ErbB family receptors occurs when ErbB3 is activated concurrently combined with either ErbB1 and/or ErbB2 receptors.^156^ When the neoplastic mammary epithelial cells were treated with 0–8 μm, in vitro, gamma‐tocotrienol suppressed the PI3K/PI3K‐dependent kinase‐1 (PDK‐1)/Akt mitogenic signaling cascade in a dose‐dependent manner.^156^

A similar in vitro study on +SA mammary neoplasm treated with combinatorial therapy consisting of a COX‐inhibitor, Celecoxib, and gamma‐tocotrienol was also reported to enhance antiproliferative effects as an improved formula with potential anti‐neoplastic properties.^155^ This treatment combination together achieved the suppression of phosphorylated Akt, NF‐kB, COX‐2, and prostaglandin levels, where the researchers described it as a new therapeutic option for breast cancer because of the antiproliferation was dependent on the dose given.^155^ Importantly, the suppression of COX‐inhibition by this formula also served to reduce the toxicity of Celecoxib, as monotherapy.^155^

Delta‐tocotrienol treatment was reported successful in reducing ionizing radiation injury in CD2FI mice (measured against a 30‐day survival post‐Cobalt‐60‐gamma irradiation) through the induction of the granulocyte colony‐stimulating factor (G‐CSF).^171^ Delta‐tocotrienol treatment had upregulated many different cytokines and proved to be exerting its radioprotective mechanism by stimulating a G‐CSF‐mediated mechanism because when the mice were injected simultaneously with delta‐tocotrienol and G‐CSF‐neutralizing antibodies, the radiation‐protectivity afforded by delta‐tocotrienol was abrogated.^171^ In addition to delta‐tocotrienol, gamma‐tocotrienol is also reported to induce radioprotection by mediating a G‐CSF mechanism.^94^ The tocopherols were known to exert radioprotection by inducing antioxidant action because the ionizing radiation damage was elicited by ROS and free radicals. Alpha‐tocopherol succinate, delta‐ and gamma‐tocotrienol have been introduced as more effective radiation countermeasures. However, delta‐tocotrienol seems to induce radioprotection by stimulating signal transduction of various molecular mechanisms and signaling pathways in addition to its antioxidant action.^171^

In summarizing the key metabolic mechanisms through which the tocotrienols broadly exert their anticancer therapeutic actions (Figure 12), they primarily involve the downregulation of the NF‐kB, PI3K/Akt/GSK, p38 (MAPK), TGF‐β, Ras/Raf/Mek/ERK, activation of the caspases and reduction in cyclin D1, cyclin‐dependent—kinases 2,4,6 and apoptosis induction.

## 3 ANTIOXIDANT AND ANTI‐INFLAMMATORY ACTIVITIES OF TOCOTRIENOL

### 3.1 Antioxidant behavior of tocotrienol

Vitamin E leads the cell antioxidant defense system, as it is a major lipid‐soluble antioxidant bound to the cellular and organelle membranes and is one that offers protection to the membranes by preventing lipid peroxidation.^172^ Antioxidant efficacy of tocotrienol is well‐documented and already established.^173^, ^174^ Oxidative stress arises from the disruption of redox‐signaling and molecular damage that erupts from endogenous free radicals such as ROS, hydroxyl free radicals, unstable superoxides, and nitric oxide (NO), released from intracellular metabolic activities and the breakdown of immune pathways.^175^, ^176^ A study conducted on evaluating the antioxidant properties of tocotrienol using primary hippocampal neurons have shown that gamma‐tocotrienol provides neuroprotection by upregulating the B‐cell lymphoma‐extra‐large (Bcl‐xL) family of proteins known to induce anti‐apoptosis and maintain neurotransmission at its optimum.^177^ The neuronal excitotoxicity was shown to release free radicals such as ROS due to post‐translational cleavage of Bcl‐xL proteins and the accumulation of its products within the mitochondrial membranes leading to mitochondrial dysfunction and depletion of intracellular ATP, resulting in subsequent neuronal death.^177^ The hippocampal neurons when treated with alpha‐tocotrienol and glutamate (which induces ROS), was found to offer neuroprotection through exerting its antioxidant properties which prevented the loss of mitochondrial membrane potential.^177^ The endoplasmic reticulum (ER)‐associated stress is mostly induced by misfolded proteins and is linked to oxidative stress because a disruption of redox homeostasis also yields an unfolded protein response.^178^ Another study investigating the anti‐ER stress mechanisms of gamma‐tocotrienol using +SA mammary tumor epithelial cells have reported that its mode of action in producing anti‐apoptotic effects is not by mitochondrial dysfunction or the death receptors but by increasing PARP cleavage and activation‐mediated protein kinase‐like ER kinase/eukaryotic translational initiation factor/activating transcription factor 4 (PERK/eIF2alpha/ATF‐4) pathway, which is a marker of ER stress response.^178^

An investigation into the impact of oxidative stress on bone formation, osteoblasts were exposed to hydrogen peroxide and were treated with varying gamma‐tocotrienol concentrations ranging from 1 to 100 μM, in which the production of malondialdehyde, a final product of lipid peroxidation, free radical release, a marker of oxidative stress, and antioxidant status in cancerous patients^179^ was dose‐dependent.^131^ Further, in evaluating the antioxidant enzyme activity, gamma‐tocotrienol prevented the reduction in superoxide dismutase (SOD, using tetrazolium assay into measuring the superoxide radicals), catalase (needed to breakdown hydrogen peroxide), and glutathione peroxidase activity (catalyzing the reduction of hydrogen peroxide and peroxide radicals) even at lower concentrations.^131^ At the higher dose, gamma‐tocotrienol itself had become toxic to the osteoblasts, suggesting that at higher concentrations, it could become pro‐oxidant or pro‐apoptotic to bone tissue.^131^ A study which had investigated the TRF‐mediated antioxidant efficacy in experimental diabetic rats had reported increased SOD activity, increased vitamin C levels, reduced lipid peroxidation in the thoracic aorta homogenates measured with malondialdehyde.^180^ Tocotrienol treatment had served to lessen the oxidative stress inflicted upon the blood vessels reiterating that the type 1 diabetes mellitus leads to atherosclerosis, resulting in vascular damage.^180^ TRF‐treated diabetic rat thoracic aorta showed reduced vascular smooth muscle cell proliferation and degeneration, a smooth lining of the intima with fewer defects, a more regular elastic lamina, squamous characteristics in the endothelium, and increased SOD activity in aortic homogenates,^180^ These results suggest that the oxidative stress and DNA damage, induced by diabetes, is attenuated by TRF treatment.^180^ Additionally, the ability of the TRF to inhibit oxidative stress arising from UV radiation in murine skin and improve wound healing mechanisms in diabetic animals by a possible free radical scavenging role has been reported.^181^

The development of diabetes mellitus also produces impaired skeletal muscle growth.^182^ A study in which diabetes was induced by a high fat diet and streptozotocin, a C57BL/6J mouse model, had shown reduced skeletal muscle‐related protein levels (4‐hydroxynonenal, protein carbonyls, Nrf2, and HO‐1), associated with oxidative damage upon treatment with TRF.^182^ These results had suggested a protective role in TRF against skeletal muscle atrophy which could arise from oxidative stress in diabetic mice, explained partly due to TRF‐induced mitochondrial biogenesis.^182^ It was achieved by the upregulation of SIRT1 (Sirtuin or silent mating type information regulation 2 homolog) 1 (*S*. *cerevisiae*), SIRT3, and AMPK pathways which aid glucose metabolism via insulin signaling in type 2 diabetic mice.^182^ Further studies are summarized in Table 2. The main strength of tocotrienols arise from their ability to influence the endogenous fat‐soluble lipids [chylomicrons, chylomicron remnants, very‐low‐density lipoproteins (VLDL), intermediate‐low‐density lipoprotein (IDL), low‐density lipoprotein (LDL), high‐density lipoprotein (HDL), cholesterol, triglycerides, and apoliproteins] preventing lipid peroxidation and attenuating oxidative stress.^183^

**TABLE 2:** The latest tocotrienol studies on oxidative stress (published in 2021)

Model	Disease	Tocotrienol treatment regimen	Results	Outcome	Reference
Strain (N2) of *Caenorhabditis elegans* (L4 stage)	Oxidative stress	100 μg/ml at 20°C for 4 h in a petri‐dish	Survival of the nematodes was considered as having antioxidant activity	Delta tocotrienol extracted from Achiote seeds possesses antioxidant properties	323
Mouse	Oxidative stress in the liver	2000 mg/kg for 14 days	Induced maximal *Nrf‐2* gene expression within 1 h.	Tocotrienols (TRF) promote antioxidant‐related hepatoprotection in mice, induced by cytoprotective *Nrf‐2* gene upregulation.	324
Sprague Dawley Rats	Oxidative stress	60 mg/kg bodyweight for 8 weeks	Increased bone strength, bone microstructure, calcium content in ovariectomized rats with antioxidant activity.	Annatto‐derived delta‐tocotrienol induces antioxidant activity and bone health in estrogen‐deficient rats.	325
Characterization of physicochemical properties of vitamin E combined with cyclodextrin (CD)	Antioxidant properties	Formation of Vitamin E and βcyclodextrin complex by solution method.	Vitamin was physically encapsulated by the β‐cyclodextrin with extended lifetime dependent on β‐CD concentration.	Antioxidant activity increased with free radical scavenging ability in the inclusion complex measured by fluorescence intensity.	326
Assessment of tocotrienol therapeutic potential in Sea Buckthorn juice.	Antioxidant, anti‐inflammatory, and neuroprotection effects	Measured by ultra‐performance liquid chromatography coupled with fluorescence detector	Anti‐enzyme and antioxidant properties of the juice correlated selectively with the composition	Valuable dietary source of vitamins E and A and which enhances antioxidant, anti‐inflammatory, and protection from neurodegeneration processes.	327
C57BL/6J WT and *db/db* mouse	Type 2 Diabetes	A modified form of tocotrienol‐β (deh‐T3β) given mixed with diet for 2 months	Improved kidney and liver function, reduced liver steatosis, faster wound healing, insulin efficiency in adipose tissue, and improved heart recovery after ischemia.	Antioxidant properties and mitochondrial function were improved.	328
C57BL/6J mouse	Obesity and obesity‐related metabolic disorders	High‐fat diet +800 mg/kg BW for 14 weeks	Lowered serum metabolites, inflammatory and antioxidant biomarkers, and improved carbohydrate metabolism.	A nontoxic dose of tocotrienol improved obesity‐associated hypercholesterolemia and hyperglycemia	196

### 3.2 Anti‐inflammatory behavior of tocotrienol

Inflammation is an integral component of innate immune reactions and comprises acute and chronic inflammatory responses.^184^ They are elicited as part of immune surveillance and host defense mechanisms for maintaining immune homeostasis.^184^ Acute inflammation is needed for prompt resolution of a disease state that is self‐regulatory while chronic inflammation is a pathophysiological defect that progresses into impaired homeostasis.^184^ Importantly, both these immune aberrant mechanisms are attenuated by tocotrienol therapy. Chronic inflammation has manifested as chronic kidney disease, rheumatoid arthritis, CVD, diabetes, cancers, neurodegeneration, metabolic syndrome, and liver disease.^185^ Tocotrienols have successfully curbed inflammation by a variety of physiological mechanisms of which the downregulation of the gene expression of pro‐inflammatory cytokines and the repression of inflammatory biomarkers are more common strategies.^186^ The prominent biomarkers that are suppressed with tocotrienols include IL‐6, TNF‐a, MDA, and C‐reactive protein although most results have been inconsistent.^187^ The dosage and the individual tocotrienol isomer profiles had reported different outcomes and in general, tocotrienols were not very successful in attenuating inflammation as per a meta‐analysis that was published recently.^184^ TRF was more effective against single isomers, of which both delta and gamma tocotrienols produced better results than alpha and beta counterparts. A dose of 400 mg/day or above had reduced MDA levels while CRP was fair downregulated.^184^ For the lack of high‐quality clinical data, the anti‐inflammatory status of tocotrienols remains inconclusive. The main concern is the discrepancy between preclinical studies and clinical studies, the former being nonreproducible in humans. There was an increase in HDL cholesterol levels following tocotrienol treatment but no effect on total cholesterol level, LDL‐cholesterol, or triglycerides.^188^ The oral administration was insufficient to upregulate tocotrienol bioavailability to repress inflammation due to shorter half‐life, fast elimination, and hindered transportation in plasma. A high quantity of dietary lipids were needed to stimulate bile secretion and emulsification for efficient absorption of tocotrienols and the cellular transportation of tocotrienols was not efficient although ready incorporation into cell membranes made cellular influx more favorable. Among the anti‐inflammatory mechanisms of tocotrienols are inhibitory action of NF‐kB and STAT‐3 pathways^189^ and suppression of inflammatory mediators such as IL‐1, IL‐6, IL‐18, IL‐8, TNF‐alpha, and inducible nitric oxide synthase.^190^ The biomarkers of oxidative stress have also shown inflammation resolution that is caused by ROS and RNS, free fatty acids, chemotherapy, and ionizing radiation. When the individual tocotrienol isomeric forms were compared, delta‐tocotrienol was more inhibitory on IL‐6 and TNF‐alpha levels in LPS‐stimulated RAW 264.6 and RAW 264.7 macrophages respectively than the gamma‐ and alpha‐tocotrienol or the TRF.^186^

Obesity and insulin resistance are two disease states in which the adipose tissue develops inflammation by the Nod‐like receptor protein‐3 (NLRP3) inflammasome activation. It leads to impaired lipid metabolism by stimulating the release of eicosanoids. Saturated fatty acid—stimulation activates the NLRP3 inflammasome in innate immune cells and thus, the gamma‐tocotrienol treatment showed attenuation of lipopolysaccharide (LPS) and SFA‐stimulated NLRP‐3 inflammasome activation in bone marrow‐derived macrophages (BMDM) in vitro.^191^ Gamma‐tocotrienol repressed the NLRP3 inflammasome activation by the downregulation of arachidonic acid metabolism (a precursor of eicosanoids), diacylglycerol, prostaglandin release, and COX‐2 activation, which led to the modulation of the BMDM lipid profile. It modulated the ensuing glycerolipid, glycerophospholipid, lysophospholipid, and sphingolipid production via the suppression of eicosanoid release and inhibited the de novo synthesis of ceramides of the dihydroceramide pathway. The lipid metabolism, which is altered during inflammation, impacts the energy homeostasis in macrophages and was shown partly rescued by gamma‐tocotrienol.^191^ The activation of the NLRP‐3 inflammasome results in aberrant innate immunity that develops into metabolic diseases, particularly type‐2 diabetes. Another in vitro study of macrophages treated with Annatto seed‐derived delta‐tocotrienol showed a marked decrease in ROS, which is the second signal needed for the priming and activation of the NLRP‐3 inflammasome.^192^ The activation of the inflammasome occurs through intracellular sensing of endogenous damage‐associated molecular patterns (DAMP), from molecules of microbial infections that produce pro‐inflammatory cytokines such as interleukin 1‐beta (IL‐1β) which is a hallmark of progression into chronic inflammatory diseases. Delta‐tocotrienol was effective in inhibiting the priming of the inflammasome by suppressing the NF‐kB pathway and the ROS production leading to the cleavage of caspase‐1 needed for IL‐1β secretion in murine macrophages, at a concentration of 1 μM that suggested its use in immune modulation (MAPK, p‐ERK, p38) and delaying of the onset of chronic inflammation.^192^ Further, the NLRP3 inflammasome activity in a chronic inflammation mimetic of type 2‐diabetes was attenuated by gamma‐tocotrienol in a model of leptin receptor knockout mice.^193^ This study introduced a two‐prong approach by gamma‐tocotrienol consisting of downregulating A20‐induced TNF‐alpha interacting protein 3‐activated NF‐kB signaling cascade and the suppression of caspase‐1 cleavage by adenosine mono phosphate (AMP)‐activated protein kinase autophagy, the mechanisms of which were successful in curtailing the progression of type‐2 diabetes in those mice.^193^

### 3.3 Link between antioxidant and anti‐inflammatory properties of tocotrienol

Cellular oxidative stress can produce chronic tissue inflammation.^194^ Antioxidant properties of a compound can also be anti‐inflammatory because the former protects the tissues from ROS‐activated tissue damage.^194^ Thus, both these metabolic pathways synergize each other. A compound which is capable of neutralizing oxidative damage, invariably serves to enhance anti‐inflammatory reactions as well.^195^ Tocotrienols are major tissue antioxidants as well as anti‐inflammatory which together produce attenuation of tissue injury that arises from oxidative agents that could continue to ignite tissue inflammation.^196^ Vitamin E forms are well‐known antioxidant nutrient supplements that act to prevent unwanted inflammatory responses that are caused by exogenous and endogenous oxidants in the cellular environment.^195^ Antioxidant agents comprise enzymatic and nonenzymatic compounds, of which, tocotrienol, a nonenzymatic compound, can induce the action of the enzymes; superoxide dismutase, catalase, glutathione oxidase, glutathione transferase, heme oxygenase‐1 Redox proteins, and peroxiredoxin.^197^ Thus, the therapeutic value of vitamin E compounds, particularly the tocotrienols is further enhanced due to its ability to stimulate dual roles by which its anti‐cancer properties are augmented.^198^ The prolonged localized cellular inflammatory reactions (generated by dysfunctional electron transport chain in mitochondria, ER and cell membrane, nicotinamide adenine dinucleotide phosphate [NADPH] oxidase, peroxidases, and nitric oxide synthase) can give way to tumorigenesis from which cancers arise, and those cancers have been attenuated by tocotrienols.^199^ Among the different types of oxidative damage, DNA oxidation, protein oxidation, and lipid peroxidation are known to produce carcinogenesis, and tocotrienols were identified as having anti‐cancer properties that ameliorated the malignant tumors by apoptosis, cell cycle arrest, and disrupted DNA replication and curbed metastasis.^199^ DNA oxidation impairs the DNA replication process, and transcription, and upregulates transversion mutations, protein oxidation will lead to peptide chain breakages and susceptibility to proteolysis, while lipid peroxidation results in cell membrane damage in all membrane‐bound organelles.^200^ Disruption of the plasma membrane would result in the inactivation of signal transduction by denaturing receptors and enzymes. The oxidative stress and chronic inflammation form an interconnected vicious cycle where both situations impinge upon the individual's immune system.^201^ The most abundant immune cell cohort that protects the organelles, tissues, and the organs from infections and injury onslaughts are the macrophages which release free radical intermediates during the process of promoting both innate and adaptive immunity.^202^ As the putative therapeutic role of the tocotrienols is fortified by its multiple roles in downregulating the oxidative, inflammatory, and cancerous responses.^203^ The mechanism of action in antioxidants is neutralizing ROS and ROS‐induced intermediates by donating hydrogen atoms, which make them potent hydrogen (or proton) donors.^204^ The antioxidant agents prevent further oxidation of ROS‐related biomolecules by participating in many rounds of peroxyl radical quenching and reduction by ascorbate or ubiquinone.^205^

## 4 TOCOTRIENOL ACTIVITY IN SYSTEMIC DISEASE

As shown in Figure 1, tocotrienols have been effective in ameliorating many diseases in preclinical and clinical studies. A plethora of research studies bears witness to the therapeutic potential of the tocotrienol isomers in health and disease. This section includes a review of experimental tocotrienol therapies that have induced beneficial effects in many human organ systems. The importance of tocotrienol as a putative therapeutic agent^206^ has been recognized with decades of research which has reported its diverse biological functions as shown in Figure 4.^207^ Tocotrienol is now well known for its antioxidant,^208^ anti‐inflammatory,^190^ anti‐cancer,^89^ immune‐modulation,^209^ damaged DNA‐repair,^210^ plasma cholesterol‐reducing,^211^ anti‐diabetic,^212^ neuro,^213^ renal^214^and skin protective^215^ effects in addition to longevity,^216^ bone health,^217^ and improved cognition.^218^ It also modulates the plasma lipid profile^219^ and the white matter lesions in the brain.^220^ The influence of tocotrienols as modulators of many cellular metabolic pathways has also been reported with the discovery of its involvement in producing cytokines,^94^ growth factors,^221^ kinases,^109^ transcription factors,^222^ adhesion molecules,^223^ and apoptotic regulators.^111^ Although tocotrienol has been widely recognized as a multi‐faceted,^224^ multi‐beneficial^225^ treatment option for many different human diseases, whether as a single therapy^92^ or in combination with other drugs,^226^ but an effective, cumulative, dose for its use has not been elucidated yet,^54^ and varies widely depending on the disease type or malignancy. The prospective recommended dosages have been described as 100–200 mg/day,^227^ for neuro and renal protection, anti‐inflammatory, and anti‐aging related diseases, but for cardio protection, it is 100–960 mg/day.^1^ The consumption of alpha‐tocopherol above 400 IU/day or about 270 mg/day is excessive or wasteful.^228^ The plasma concentration of the tocotrienol forms alpha, gamma, and delta are considerably increased in the fed‐state with a two‐fold greater absorption.^229^ Double dosing or the same recommended dose taken twice daily had appeared effective since tocotrienol has a short half‐life and such a regimen serves to increase the circulating plasma level of those tocotrienol forms compared to tocopherol.^230^

### 4.1 Renal disease

Renal disease can occur as an adverse effect of anti‐cancer treatments^231^ as well as other drugs prescribed for a multiarray of human diseases.^232^ Acute kidney failure could develop within a short time or because of aging or chronic disease conditions.^233^ In this section, the therapeutic potential of tocotrienol is discussed relevant to renal dysfunction, which has been a promising treatment strategy to restore renal homeostasis.^234^, ^235^ An imbalance of the steady‐state in renal functionality could evolve due to the toxicity of different pharmacotherapy or overloading of various metabolic compounds or drugs given to alleviate ill health that is excreted by renal ultrafiltration. Renal nephropathies are a combination of several disease conditions associated with the kidneys and may include, kidney stones, analgesic abuse, multiple myeloma, untreated urinary tract infections, glomerulopathies, non‐diabetic kidney disease, and hypertension.^236^, ^237^

A randomized, double‐blinded, placebo‐controlled clinical trial was conducted in patients having diabetic nephropathy (usually a complication of diabetes which requires either a renal transplant or dialysis), and were supplemented with tocotrienol (as Tocovid, 200 mg twice daily for 12 weeks).^238^ Tocotrienol treatment had produced a remarkable decrease in serum creatinine and a marked increase in estimated glomerular filtration rate (eGFR; which are common biomarkers of end‐stage renal complications).^238^ These biomarkers had remained static for 6–9 months post‐treatment and other renal dysfunction parameters such as serum HBA1C, MDA, TNF‐alpha, VCAM‐1, and urine albumin and creatinine ratio had not been reduced.^239^ This trial was limited to hyperglycemic patients having type 2 diabetes and those receiving treatment for cancer, cardiovascular, and liver disease, and those taking vitamin C and E, polyphenols, or glutathione were excluded.^239^ Moreover, as the serum tocotrienol bioavailability diminishes after around 2 months from treatment, and is mostly deposited in adipocytes, any measurement relating to its tissue retention must be made by sampling the adipose tissue.^239^

The Phase II trial of the previously mentioned study [that sampled individuals diagnosed with later stages of Diabetic Kidney Disease (DKD)] had Tocovid administered for 16 weeks, was extended to 12 months of twice‐daily supplementation of 200 mg of tocotrienol as another separate study.^18^ The treatment‐washout period (when a participant is taken off a study drug or other medication to eliminate the effects of the treatment) remained up to only 6 months with no improvement in serum creatinine level or eGFR.^18^ However, up to 2 months post‐treatment, serum creatinine levels remained reduced and the eGFR increased, without a significant improvement in the urine albumin to creatinine ratio (UACR).^18^ This investigation was hampered by the Covid‐19 situation, but testing had continued every 2 months during the 12‐month trial duration. The statistics on DKD have revealed that up to 40% of diabetic patients are likely to develop DKD and most of those suffering from DKD present up to 50% of end‐stage renal disease.^18^

Renal injury is induced by both hyperlipidemia and hyperglycemic conditions acting synergistically with each other, and patients with diabetes mellitus are highly prone to developing diabetic nephropathy as dyslipidemia, a high increase of fats and cholesterol accumulation in blood, which commonly occurs in those individuals.^240^ A study that explored the efficacy of the TRF as a treatment offered for nephroprotection, had evaluated the expression of TGF‐β on fibronectin and collagenase IV levels in kidney tissue of type‐2 diabetic rats.^240^ The outcome was the attenuation of kidney injury, after continued treatment for 16 weeks.^240^ This study demonstrated that the TRF acts as a potent hypolipidemic, hypoglycemic, and an antioxidant agent which protects against the development of diabetic nephropathy in rats.^240^

The chromium compounds, specifically those of chromium IV poses a high risk of developing acute kidney injury (AKI) through industrial or occupational exposure, or by inhalation, ingestion, or intravenous administration, in both humans and animals.^241^ A study on male Wistar rats in which AKI was induced by potassium dichromate injection and treated with TRF (with 200 mg/kg daily for 3 weeks) to investigate the nephroprotection offered by tocotrienol, reported sustainable glomerular function, redox status, and selective reabsorption in the proximal convoluted tubule.^242^ The untreated group developed AKI within 48 hours with significant oxidative stress‐induced tissue damage also validated by kidney histology.^242^ Prolonged chronic renal disease is known to be induced by hyperlipidemia, which also causes atherosclerosis. An animal study using Wistar rats were treated with the TRF (extracted from palm oil) after inducing atherosclerosis by feeding an atherogenic diet consisting of high cholesterol, cholic acid, and beef tallow having high calorific value.^243^ After 6 weeks of TRF treatment (given 100 mg/kg body weight), the animals displayed significant improvement in kidney function including eGFR.^243^ The untreated controls had developed dyslipidemia, as well as atherosclerosis and the researchers, concluded that the TRF offers nephroprotection against an atherogenic diet‐induced progressive chronic renal dysfunction in Wistar rats.^243^

A study done on the dietary intervention of vitamin E in chronic hemodialysis patients given a combination of tocotrienol (TRF, 180 mg/daily) and tocopherol (40 mg/day) reported a significant lowering of the lipid profiles after a 16‐week supplementation.^244^ At this end‐stage, renal functioning showed no response to either inflammatory markers (IL‐6 and C reactive protein) or antioxidant markers (MDA and total antioxidant power) for the duration of this study.^244^ The TRF intervention group displayed higher apolipoprotein A1 (the main lipid component in high‐density lipoprotein (HDL) or good cholesterol and a biomarker of lipid metabolism) and cholesteryl‐ester transfer protein activity which is a plasma protein bound to HDL, that transports cholesterol).^244^

### 4.2 Cardiovascular disease

Cardiovascular disease (CVD) is considered as the number one global cause of mortality with around 18 million persons dying each year from it.^245^ CVD risk factors are multifocal and high lipid profiles, lead to atherosclerosis and other vascular defects which arise commonly in most populations that consume high fat (lipid peroxidation, dyslipidemia) and/or high sugar diet.^246^ These defects arise from mitochondrial glucose oxidation resulting in mitochondrial ROS, which causes oxidative stress damage to the vasculature.^247^ The superior benefits of tocotrienol treatment in lowering CVD are established because of its pharmacotherapeutic actions such as the inhibition of vascular smooth muscle proliferation,^248^ HMG‐CoA reductase activity, which is the major rate‐limiting enzyme in cholesterol biosynthesis,^249^ oxidation of low‐density lipoproteins (LDL),^206^ monocytes endothelial cell adhesion,^250^ downstream inhibition of protein kinase C (PKC)^251^ and platelet aggregation.^252^ However, there are some studies which have reported not having either superior or detrimental effects upon tocotrienol supplementation in CVD, possibly due to insufficient bioavailability,^253^ improper delivery (oral gavage being a reason for sub‐optimal accumulation and tissue retention)^254^ and poor metabolism.^48^ Also, atherosclerosis results as a response toward vascular injury caused by hyperlipidemia that progresses into hypertension.^255^

The CVD risk increases with aging, particularly in the above 70‐year category.^256^ The common treatment available for those aged individuals is the use of statins to lower cholesterol levels^257^ and beta‐blockers to control hypertension.^258^ However, these medications are prone to side effects, such as cardiomyocyte thickening^259^ atrial or ventricular hypertrophy of the heart walls and wasting muscle diseases (such as rhabdomyolysis), muscular pain, and soreness.^260^ Therefore, tocotrienols have been experimented with by many researchers, given its antioxidant, anti‐inflammatory, and anti‐diabetic properties. Those individuals contracting diabetes run a very high risk of presenting with CVD symptoms, particularly vascular injuries.^261^ The onset of aging can create a vicious cycle of diseases such as diabetes, renal disease, and CVD which are interdependent and the therapeutic potency of tocotrienol is that it consists of nutraceutical properties which could alleviate many metabolic deficiencies (Figure 13).^262^, ^263^

Varying concentrations of alpha‐, gamma‐, and delta‐tocotrienol forms in combination with alpha‐tocopherol and Tocomin, a patented self‐emulsifying tocotrienol and tocopherol complex having better absorption as a commercially available dietary supplement, were tested for their efficacy in inducing antioxidant functions in a pyrogallol‐induced oxidative stress condition in an in vitro study of the rat thoracic aorta.^264^ The inhibition of the superoxide generated by the treatment combinations used were measured by the hypoxanthine/xanthine oxidase assay and in the presence of NADPH oxidase, a potent generator of ROS.^264^ This study reported that Tocomin had a superior antioxidant effect followed by alpha‐tocopherol than the other tocotrienol forms on vascular function and for optimal antioxidant activity in the aorta, a combination of both tocotrienol and tocopherols was needed. The same study also measured the endothelium‐mediated relaxation induced by oxidative stress in the presence of acetylcholine (known to induce endothelium‐dependent relaxation) and sodium nitroprusside (a vasodilator which induces endothelium‐independent relaxation) in which Tocomin was 100 times more effective than alpha‐tocopherol alone or when the different tocotrienol forms taken individually.^264^ This research confirmed that the tocotrienols together act as a cardioprotective agent against oxidative stress‐induced vascular dysfunction.^264^ Importantly, vitamin E was found to increase nitric oxide (NO) production which in turn serves to improve the endothelial‐dependent vasodilatation by relaxing the vascular smooth muscle cells.^265^ NO is produced by the enzyme l‐arginine‐mediated by eNOS which is beneficial in CVD.^266^ NO at low levels is known to neutralize free radicals to improve oxidative stress damage in blood vessels.^266^

### 4.3 Nervous system disease

The beneficial role of vitamin E in neuroprotection is already recognized.^267^ The importance of tocotrienols is established given its ability to maintain neuronal membrane integrity (being lipophilic in nature) by counteracting the ill effects of lipid peroxidation.^268^ Orally delivered tocotrienols reach brain tissue^30^ and cerebrospinal fluid^269^ through systemic circulation and protect against free radical‐induced oxidative damage leading to neuronal death and defective synapses.^270^ This process has been validated by its neuroprotective action against glutamate‐induced ROS, NO, and/or neurotoxic metabolites.^271^ Tocotrienol supplementation was very effective in sustaining the structure and function of the nervous system.^272^ Tocotrienol consists of properties which can nullify the exposure to damaging neurotoxic metabolites produced within the body and significantly attenuating subsequent neurodegeneration which impairs cognitive functions.^213^, ^218^ Although the mode of action of tocotrienols in neurology is still largely unknown, it is deemed beneficial to maintaining the health and prevent diseases of the nervous system.^273^ Tocotrienol treatment of HT4 cells, which are mouse neurons taking the properties of differential neurons at restrictive temperatures such as 39 Celsius after glutamate challenge, was promising although it is speculated as acting along with an antioxidant‐independent mechanism.^274^ These may include stimulating other signal transduction pathways leading to the modulation of cytokine production.

The cerebral small vessel disease is a hitherto unexplored neurological condition which runs a high risk for the development of several debilitating neuropathies such as ischemic stroke, dementia (up to 40% risk in older people), Alzheimer's disease, and acute physical disabilities (mostly in the over 65 ages).^275^ It occurs because of perforations in the blood vessels in the brain which is concurrent with the inability to self‐regulate the cerebral blood flow.^275^ These hemodynamic changes are produced by hypertension and arterial stiffness caused due to oxidative stress which arises in the cerebral vasculature and when treated with the vitamin E derivatives (alpha‐tocopherol and alpha‐tocotrienol), was significantly downregulated.^275^ Vitamin E is a beneficial treatment for neuropathological conditions because of its ability to protect PUFA which offers neuroprotection to the brain.^22^ Arachidonic acid is the major PUFA present in the central nervous system (CNS) and is metabolized by both enzymatic and nonenzymatic pathways, which produce neurotoxic metabolites.^276^ Neurotoxicity was attenuated by nanomolar concentrations of palm oil‐derived alpha‐tocotrienol, thus preventing neurodegeneration.^277^ The effectiveness of tocotrienol forms was tested in microglia that are the most abundant, (up to 80%) resident immune cells of the CNS, which respond to injury and stress by releasing NO at toxic levels to the brain tissue.^278^ Mouse BV2 microglia were tested in vitro by adding different concentrations of three palm oil‐extracted tocotrienols (alpha, gamma, and delta) to examine the NO release induced by lipopolysaccharide (LPS).^278^ The results revealed a significant lowering of NO production by the microglia treated with the different tocotrienol forms (at 48 h LPS posttreatment) and delta‐tocotrienol was the most effective at a 50 μM concentration. None of the isomers was found to compromise the microglial viability.^278^

Chronic cerebral hypo‐perfusion (known to set in with aging) develops low cerebral blood flow which results in neuronal cell death leading to neurodegeneration of cognitive functions.^277^ A Sprague–Dawley rat model of similar neurodegeneration was induced by bilateral ligation in two carotid arteries to lower the blood flowing into the brain and were named the two vessel‐occluded (2VO) group. These mice were compared with a Sham group and a vitamin E tocotrienol‐treated group in which the oxidative stress levels were ascertained by measuring the isoprostane level in brain homogenates, which is a prostaglandin‐like accurate marker of lipid peroxidation in both animal and human models of oxidative stress in the CNS.^277^ Neurodegeneration status was assessed by counting the dead neurons in the hippocampus, cerebral cortex, white matter, and the visual system. There was no significant difference either in the isoprostane levels or the neuronal death count between the vitamin E tocotrienol‐treated group and the sham group.^277^ It provided evidence that tocotrienol offers neuroprotection in conditions of insufficient cerebral blood flow that reduces the ATP supply to the brain.^277^

Parkinsonian disease (PD) is a progressively debilitating neurodegenerative pathologic condition induced by a marked reduction in the dopaminergic neurons which causes motor and nonmotor dysfunction.^279^ In a recent study (in 2021), PD was induced by a single dose of intracisternal injection of 6‐hydroxy dopamine (6‐OHDA) in Sprague–Dawley rats, and the efficacy of tocotrienol was measured utilizing behavioral parameters such as paw retraction, beam travel, and cylinder tests and immunohistochemical specimens obtained from the substantia nigra in the mid brain. The diseased animals showed depigmentation, loss of dopaminergic neurons, and striatum fiber density.^279^ and alpha‐tocotrienol showed amelioration of the symptoms of PD and was able to reverse the neurodegeneration also by improving motor deficiencies.^279^

The multi‐drug resistance protein‐1 (MRP‐1) is a key factor associated with the induction of ischemic stroke in mice and was found elevated in glutathione‐induced mouse brain lesions.^280^ Alpha‐tocotrienol was identified as a neuroprotector which shields against stroke in vivo, by suppressing the neurotoxicity that arises from glutathione depletion and microRNA activity in the areas of brain affected by stroke.^280^ Primary cortical neurons challenged with glutathione oxidase in vitro, showed reduced ischemic stroke lesions when treated with alpha tocotrienol, but its mode of action was attenuated in MRP‐1‐knockout mice.^280^ Thus, MRP‐1 also appears to play a protective role in downregulating neuronal cell death by increasing glutathione efflux from neural cells and it was suggested that the therapeutic action of alpha‐tocotrienol may be mediated via a novel mechanism involving MRP‐1 which makes stroke‐related MRP‐1 and microRNA as sensitive targets of tocotrienol.^280^ Further, the elevation of intracellular glutathione disulfide (GSSG, the oxidized form of glutathione) triggers neural cell death. Excessive cellular GSSG is toxic and therefore pumped out at the expense of ATP. Inefficient clearance of intracellular GSSG was shown to build‐up neurotoxicity. These observations highlight the significance of cellular GSSG clearing systems in neuroprotection, especially in the context of the stroke where oxidative stress is overt.^281^

White matter lesions in the brain are related to cerebral small vessel disease. In evaluating tocotrienol treatment on white matter lesions, twice daily supplementation of an oral tocotrienol mix, was significantly effective compared to the control group.^220^ It was concluded that the tocotrienol therapeutic potential in neuropathies considerably increases when supplied as a mix of the different forms than single isomer supplementation.^220^ Their conclusion was further supported by studies carried out with Tocomin, which displayed superiority against treatment with individual tocotrienol forms since it consists of a mix of both tocotrienols and tocopherols.^282^ The superiority of tocomin compared to those individual vitamin E forms was reported in impaired cellular antioxidant behavior. It may be attributed to the release of nitric oxide (NO), less eNOS and NADPH oxidase (Nox‐2) activity, activation of diacyl glycerol, and subsequent protein kinase C (PKC). Less NO leads to accumulation of superoxide radicals that increase oxidative stress resulting in impaired cellular functions.^264^

Hyperalgesia is extreme sensitivity to pain, and results from damaged nerves and/or chemical changes in nerve pathways and also is associated with microvascular complications of diabetes mellitus.^283^ This excruciating pain is related to oxidative and nitrosative stress arising from ROS, NO, cytokines, and apoptosis that is referred to as diabetic neuropathic pain and was induced in an experimental diabetic rat model to investigate the effectiveness of tocotrienol treatment in alleviating the pain.^283^ Those rats were treated with tocotrienol combined with insulin, from which the hyperglycemia was improved with a marked reduction in pain by modulating the oxidative and nitrosative stress factors, caspase 3, and proinflammatory cytokine release.^283^

Alpha‐tocotrienol is well known for its neuroprotective effects in ischemic stroke.^284^ In a canine model of ischemic stroke treated with tocotrienol‐enriched fraction for 10 weeks, the fluoroscopy‐guided angiography images and magnetic resonance imaging of the brain in the intervention group revealed improvement in the areas of stroke attributed to sustained collateral cerebrovascular circulation in the ischemic territory.^284^ Tocotrienol enrichment showed upregulation of the tissue inhibitor of metalloprotease‐1 (associated with vascular remodeling) and downregulation of matrix metalloproteinase‐2 which is a biomarker of neural inflammation, the breakdown of the blood–brain barrier, and degeneration of the myelin proteins.^284^ Hence these results warrant further investigation to assess the effectiveness of tocotrienol in patients presenting transient ischemic attacks and those persons are at high risk of developing severe stroke.^276^, ^284^

The medicinal efficacy of tocotrienol is strengthened by its ability for inducing hypolipidemia and reducing plasma cholesterol levels.^285^ A low concentration of tocotrienol (between 2 μM gamma‐tocotrienol giving 50% inhibition and 10 μM giving 80% inhibition) is deemed sufficient to suppress the activity of the hepatic enzyme, HMG‐CoA reductase (3‐hydroxy‐3‐methyl‐glutaryl‐coenzyme A reductase/HMGCR) required for cholesterol synthesis in the liver via the mevalonate pathway.^286^ Furthermore, the delta and gamma forms of tocotrienol are involved in the sterol‐regulated degradation of proteins through the ER membrane‐associated proteins such as insulin‐induced genes (Insigs) that activate the degradation of misfolded and dysfunctional proteins via ubiquitin ligases. It is also linked to the regulation of the HMG‐CoA reductase activity in a complex feedback loop.^287^ Similarly, a nanomolar concentration of gamma and delta forms of tocotrienol were found to be an order of magnitude higher than what was required to protect the neurons from neurotoxic insults, as per the data from cell culture studies.^288^ One drawback of tocotrienol metabolism is that it has low bioavailability when taken through the oral route.^54^ Among the cohort of tocotrienol isomers, alpha‐tocotrienol can suppress neurodegeneration of nerve cells at nanomolar concentrations by regulating specific mediators which induce cell death and oral supplementation of tocotrienol is known to protect against the adverse effects of stroke.^289^

### 4.4 Lipidomic disease

As indicated by previous studies, tocotrienol transportation within tissues and all vital organs has been described, where it had reached the skin, liver, heart, spinal cord, skeletal muscles, lung, adipose tissue, and brain even when consumed as an oral supplement.^290^, ^291^ Additionally, tocotrienol was detected in measurable amounts in new‐born rat tissues when pregnant rats were orally administered.^292^ The absorption of tocotrienol takes place in the small intestine where it enters the systemic circulation first as chylomicrons which later breakdown and attach to circulating lipoprotein carriers for transportation into cells.^293^ A common method that enhances fat‐soluble vitamin absorption is to deliver it in the form of an emulsion, and ingested tocotrienol forms an emulsion with the gastrointestinal juices as soon as it enters the intestine.^294^ However, there is evidence that when both tocopherol and tocotrienol are digested together, the tocopherols outcompete tocotrienol during absorption, which suggests that it is prudent not to consume both these vitamin E forms, at the same time.^295^ Tocopherol cellular uptake and transport conventionally occur through binding with tocopherol transfer protein (TTP) and it is extended to the cellular uptake of the tocotrienols as well.^296^ It is also known that tocotrienol absorption is enhanced when taken together with food, such as after a meal in healthy individuals.^297^ Tocotrienol absorption is deemed dependent on the intake of food, the lipid content of food, and bioavailability.^298^

Obesity is considered as a pathological condition due to excessive fat deposits in the adipocytes arising from defective lipid metabolism and glucose homeostasis.^196^ Obesity is also characterized as being a reservoir of signaling molecules involving cytokines, chemokines, oxidative stress, and inflammatory biomarkers and consequently is prone to persisting chronic low‐grade inflammation in fatty tissue.^196^ Tocotrienols have reported efficacy in attenuating obesity and was validated by an investigation on the impact of tocotrienol supplementation in high‐fat diet‐induced obesity in mice.^196^ It revealed beneficial changes in serum metabolites related to multiple metabolic pathways which modulated oxidative stress, lipid peroxidation, metabolism of fatty acids, amino acids, phospholipids, sphingolipids, and the gut‐inhabitant microbiome in obese mice, supplemented with tocotrienol.^196^ A comparison between high‐fat diet‐fed obese mice with a low‐fat diet‐fed counterparts, supplemented with delta‐tocotrienol revealed attenuation of fat deposition in adipocytes (being smaller in size), elevated anti‐inflammatory adipokines, and low infiltration of macrophages into adipose tissue.^299^ Delta‐tocotrienol supplementation had reduced the markers of fat oxidation and fatty acid synthesis in adipose tissue and liver.^299^ The outcome of this study indicates that tocotrienol supplementation promotes metabolically healthy status in obese mice which could be extended into clinical studies.^299^

### 4.5 Skin disease

Atopic dermatitis treated with gamma‐tocotrienol combined with alpha‐tocopherol was delivered to the skin as a nano‐emulsion entrapped in nanoparticles enhanced by microwave exposure [pretreatment by microwave (1 mW/3985 MHz). This treatment promoted therapeutics accumulation in the epidermis through enhancing nano‐emulsion penetration into the skin, which was successful in attenuating the disease.^300^ The available standard therapy for dermatitis consists of steroids and other immunosuppressive drugs which produce side effects and gamma‐tocotrienol administered via the oral route is deemed ineffective due to its low bioavailability, in the epidermis and dermis.^300^ This delivery mechanism presents a de novo treatment modality in skin diseases because targeted delivery of gamma‐tocotrienol to the skin had proven to be more effective in alleviating dermatitis symptoms.^300^ Wound healing is yet another function of tocotrienol treatment.^301^ Mice exposed to deep‐partial thickness burns were treated with TRF as a cream combined with epidermal growth factor (EGF) and macroscopic and microscopic images of the skin were analyzed along with tests on biochemical parameters.^301^ The EGF and TRF formulation reduced neutrophils, lymphocytes, and myofibroblasts with no change in adipocytes.^301^ Additionally, lowering of lipid peroxidation and nitrite levels were observed indicating reduced oxidative stress. TRF together with EGF presents a new therapeutic combination in the treatment of burn wounds.^301^

### 4.6 Glycemic disease

In a clinical study of type‐2‐diabetes, delta‐tocotrienol administered concurrently with oral glycemic therapy reported a significant reduction in the glycemic condition, an increase in plasma insulin, and insulin resistance alongside the attenuation of markers related to oxidative stress and inflammation. The expression of a repertoire of micro ribonucleic acid (miRNA are short, noncoding RNAs, which repress the synthesis of certain proteins by post‐transcriptional destabilizing of mRNA) is indicative of glycemic control in which some were elevated (miRNA‐126, 132) and some were downregulated (miRNA‐375, 34a, and 21).^302^ Thus, the glycemic therapy synergized by delta‐tocotrienol has proven its ability in ameliorating long‐term type‐2 diabetes in patients without causing adverse effects.^302^

### 4.7 Lung disease

Asthmatic Norway brown rats were treated with synthetic anti‐asthmatic drugs with either one of adjunct tocotrienol, carotene, or dexamethasone post‐challenge with ovalbumin, to examine the therapeutic outcome of tocotrienol in allergic asthma.^39^ Allergic asthma is a chronic airway inflammatory disease which compromises respiration and its synthetic drugs exhibit multiple adverse side effects.^39^ The groups treated with either tocotrienol or carotene, which are natural phytochemical nutrients, reported favorable outcomes in alleviating asthmatic symptoms in this rat model.^39^ The observations were supported by decreased; rat plasma C‐reactive protein, immunoglobulin E, and the absence of histologically validated immune cell infiltration in bronchial walls and edema, which signified healthy lungs.^39^

Emphysema is a chronic obstructive pulmonary disorder (COPD) that occurs partly due to inflammation and oxidative stress.^303^ The standard treatment offered are corticosteroids which do not alleviate oxidative stress and some patients become steroid‐resistant.^303^ Since the tocotrienols are antioxidant and anti‐inflammatory agents, a study in which emphysema was induced by exposure to cigarette smoke in BALB/c mice, reported attenuation of disease when given gamma‐tocotrienol compared to prednisolone, a steroid.^303^ Gamma‐tocotrienol was effective in ameliorating emphysema lesions of murine lungs, reduce bronchial wall thickening, pulmonary pro‐oxidant, and pro‐inflammatory gene expression, biomarkers of oxidative stress and inflammation which also restored the normal lung antioxidant capacity.^303^ This study provides evidence of tocotrienol having a therapeutic role in COPD treatment in a mouse model.

### 4.8 Eye disease

The development of cataracts in the eyes is a leading cause of visual impairment which arises as an early‐onset complication of diabetes.^304^ There are studies that have reported the cataract‐inhibiting properties of tocotrienol such as reducing lens stress and inhibiting cataract‐genesis in mice fed a galactose‐rich diet.^305^ A third study in which diabetes was induced by streptozotocin injection reported curtailment of cataract progression in the delta‐tocotrienol‐treated group compared to the control group. Topical tocotrienol application was capable of suppressing; the NF‐kB pathway, inducible nitric oxide synthase (iNOS) activation, and oxidative‐nitrosative stress, while improving ATP synthesis and ATPase activity, restoring lens proteins, and calpain activity as well as reducing lens aldose reductase enzymatic activity.^304^

### 4.9 Bone disease

Osteoblast migration is needed for bone remodeling and repairing the fractures in support of which delta‐tocotrienol has been identified as a potential source of osteoanabolic mechanisms.^306^ This study delineated a role for tocotrienol therapy in human bone marrow mesenchymal stem cells exerting its influence through the mediation of Akt phosphorylation and the activation of the Wnt/beta‐catenin pathway.^306^ Tocotrienols strengthen the structure and functions of the bone mass in the skeletal system.^306^ Delta‐tocotrienol was shown to stimulate beta catenin‐regulated osteogenic targeted genes involved in osteoblast differentiation and migration.^306^ Therefore, delta‐tocotrienol plays a vital role in maintaining bone health by reducing bone loss by inhibiting osteoclast formation and bone resorption through the modulation of the Receptor activator of nuclear factor‐kappa‐Β ligand (RANKL) and osteoprotegerin (OPG) expression.^307^ Another study investigating the bone properties in obese mice treated with annatto‐oil‐extracted tocotrienol combined with green tea polyphenols displayed increased bone volume through the modulation of the gut microbiome composition and the biosynthesis of vitamin K2.^308^ Thus, it presents a link between bone health maintenance and the gut‐inhabitant microbiome which could be a novel diet‐modulated therapy in promoting bone vitality in obesity models.^308^

Rheumatoid arthritis (RA) is a systemic and autoimmune disease of the joints, which causes severe joint pain, bone erosion, and cartilage destruction stimulated by inflammatory mediators.^309^ RA was induced in Dark Agouti rats by collagen type II intradermal injection and were treated with the TRF.^309^ Compared to the group without TRF, the intervention group displayed significant attenuation of collagen‐induced arthritis in this in vivo rat model by reducing the expression of inflammatory mediators and joint destruction.^309^

## 5 RAMIFICATIONS OF THERAPEUTIC USE

Although this review has focussed on the beneficial effects of Tocotrienols, negative outcomes have also been reported, although in‐depth analysis of these studies is beyond the scope of this review. Notwithstanding the large amount of experimental data that is published on the beneficial characteristics of the different forms of tocotrienol, negative outcomes have been reported in certain studies. Dairy animals treated with gamma‐tocotrienol had recorded a loss of endothelial barrier integrity in bovine mammary endothelial cells at lower concentrations without displaying adverse effects on cell viability in other cell types at like concentrations. The impact of antioxidant therapeutic effect of vitamin E forms is important during calving and peak lactation which otherwise produce inflammation and destruction of macromolecules (lipids, nucleic acids, and proteins) leading to the manifestation of immune dysfunction and metabolic disease. The same study also showed superior antioxidant effects of the tocopherols compared to the tocotrienol counterparts.^310^ The metabolism of both tocopherol and tocotrienols undergo a similar process although the tocotrienols degrade in a more complex manner. In vitro differences have been observed in a HepG2 cell culture model although it was postulated to be different in in vivo systems. Thus, the metabolism and biodegradation of these two cohorts are correlated to their bioavailability and retention within the cells which showed less accumulation in the case of tocotrienols making the tocopherols have a more therapeutic effect.^311^ Other instances of tocotrienols being inferior to its tocopherol forms is having low plasma bioavailability in cells when consumed through the oral route. It clearly is an impeding factor in the tocotrienol therapeutic spectrum. Further, tocopherol bioactivity in the cellular system is augmented by the hepatic plasma tocopherol transfer proteins and tocopherol‐associated proteins, both of which assist in the transportation of the tocopherol forms whereas the tocotrienols have less affinity towards those carrier‐mediated cellular influx mechanisms.^312^ Tocopherols and tocotrienols display unique concentrations in various tissues as per a study that quantified the vitamin E forms in various mouse tissue. The study using high‐pressure liquid chromatography (HPLC), had reported the existence of a selective mechanism that is based on the antioxidant capacity of the toco family forms although it is debatable. This study utilized hairless mouse tissue to quantify these lipophilic antioxidants that recorded almost 99.8% of alpha‐tocopherol in the brain with no significant presence of tocotrienols. The overall quantification showed under 1% tissue tocotrienol whereas most other tissues had considerably high percentages of the tocopherol forms.^313^ The biopotency of tocopherols is greater than that of tocotrienols and is a measure of the biological half‐life, which is longer in tocopherols. Thus, tocotrienol degradation is faster with it being metabolized quicker than the tocopherols. This selective metabolism is based on the activity of the human cytochrome P450 also known as (CYP4F2) which mediates the ω‐hydroxylation step that converts both tocopherols and tocotrienols to carboxychromanol, where CYP4F2 is considered the only enzyme that catalyzes the metabolic breakdown of intracellular vitamin E forms. This degradation step is determined by the respective molecular structures of the vitamin E forms and depends upon the stereochemistry of the sidechain and the number of methyl groups present in the chromanol ring structure. Thus, tocotrienols are metabolized and excreted in urine faster than tocopherols because of the unsaturated isoprenoid sidechain composition.^314^

## 6 FUTURE DIRECTIONS

There are many avenues along which tocotrienol research could be guided in the future to gain clinical benefits from tocotrienol therapy. Thus, continued research is encouraged that focuses more on the clinical therapeutic potential of tocotrienol in ameliorating the inflammatory diseases for which it had exhibited experimental efficacy. One drawback in tocotrienol research is lacking clinical models possibly due to needing invasive techniques for investigations. Most of the research has been conducted with in vitro studies that do not depict the in vivo conditions. There are extensive studies performed on microRNA and small interfering RNA‐involving cytolytic mechanisms which do not represent the clinical picture. A gap in the present tocotrienol research still prevails because the means to elicit a long‐lasting therapeutic effect has not been successful. Interestingly, tocotrienol possesses strong inhibitory anticancer properties which could be the highlight of its therapeutic spectrum. A detailed assessment of the drug–drug interactions has not been conducted with tocotrienol therapy in cancer patients, because the cancer physiology varies significantly among the cancer types. The cancer studies have so far concentrated on adjuvant tocotrienol therapy in attenuating the cancers although the ability of tocotrienol to overcome the toxicity of chemotherapy is still lacking. Since tocotrienols are plant‐derived phytochemicals, there are abundant resources from which these compounds could be extracted. Because certain studies have shown dose‐dependent activity in tocotrienols, new methods are needed for obtaining extract, with an extended half‐life, and to overcome the side effects. To enhance its therapeutic efficacy, the bioavailability threshold and duration of retention should be increased. These properties could be enhanced by combining with appropriate adjuvants that can be identified by analytical computer software which matches membrane‐bound receptors and their docking compounds (such as CADD).^315^

## 7 CONCLUSIONS

This review has summarized the salient aspects of experimental tocotrienol therapy combined with the latest findings that focused on modulating the immune‐metabolic responses in different disease states, particularly, cancer. The superiority of tocotrienols in disease treatment (Figure 13) compared to tocopherols is already established. Tocotrienols are considered superior to the tocopherols for many reasons that are associated with biological functions related to health and disease. Alpha‐, gamma‐, and delta‐tocotrienol forms are identified to be more effective than alpha‐tocopherol which was more commonly experimented with during the early days of its discovery. More recent research studies have revealed that tocotrienols surpass tocopherols by being better antioxidant, antihypertensive, anti‐cancer metastatic, anti‐inflammatory, anti‐hypercholesterolemic, anti‐atherogenic, anti‐tumor, anti‐proliferative, apoptotic, anti‐angiogenic, and neuroprotective therapy that are documented through a large number of preclinical and clinical studies. The preferential uptake of topically applied tocotrienols into ocular tissues, maintaining normal bone calcification in rats, better therapeutic effects in obesity, diabetes, and osteoporosis have been documented. The maintenance of tocotrienol levels up to 24 h after intragastric administration was documented in adipose tissue of epididymis, renal, subcutaneous, and brown fat tissue. The highest concentration of tocotrienols was reported at 8 h in serum, liver, mesenteric lymph nodes, lungs, and spleen in rats. Tocotrienols are transported within membranes and incorporated into membranes more than tocopherols in rats. There is paucity of information in identifying a biochemically, physiologically, and immunologically active, formulation to overcome its deficiencies as a therapeutic agent that have been reported by certain other studies such as having low bioavailability, low tissue penetration efficiency except in skin, short half‐life, and higher drug efflux from cells. The protective role of tocotrienol on PUFA could be employed in mediating anti‐oxidation of dietary fatty acids and regulating endocytosis and exocytosis via ER. Those mechanisms act to restrict or enhance drug influx or efflux, thus augmenting drug metabolism and increasing chemosensitivity to curative compounds. Importantly, tocotrienols can cross the blood–brain barrier in restoring cognitive functions in neurodegenerative diseases which warrants further investigations.

## AUTHOR CONTRIBUTIONS

Concept, writing, and organizing by Ranmali Ranasinghe. Constructive criticism, Guidance, and Editing by Anthony Zulli and Michael Mathai.

## FUNDING INFORMATION

No funding was received to assist with the preparation of this manuscript.

## CONFLICT OF INTEREST

The authors have no conflicts of interest to declare that are relevant to the content of this article.

## Data Availability

Data sharing not applicable to this article as no datasets were generated or analysed during the current study.
